# Supplementary material for: Development of a RIPK1 degrader to enhance antitumor immunity
Source: Nat Commun. 2024 Dec 16;15:10683. doi: 10.1038/s41467-024-55006-2 (PMC11649918; doi:10.1038/s41467-024-55006-2)
Supplement: Supplementary file 1 — Supplementary Information [file 41467_2024_55006_MOESM1_ESM.pdf]

Supplementary table 1: Absorption, distribution, metabolism, excretion, and toxicity (ADMET) summary of LD4172.

***In Vitro* Metabolic Stability**	Human Liver S9	$T_{1/2}$ (mins)	21.1
		CLint ($\mu L \cdot min^{-1} \cdot mg^{-1}$ protein)	32.8
	Mouse Liver S9	$T_{1/2}$ (mins)	9.7
		CLint ($\mu L \cdot min^{-1} \cdot mg^{-1}$ protein)	71.6
	Human Hepatocytes	$T_{1/2}$ (mins)	56.3
		Predicted CLint ($mL \cdot min^{-1} \cdot kg^{-1}$)	15.6
**hERG Inhibition**		% Inhibition @ 30 μM	4.85

***In Vivo* pharmacokinetics**	C57BL/6J mice (i.v. 1mg/kg)	$T_{1/2}$ (h)	3.3
		C_{max} (μM)	6.3
		$AUC_{0\sim\infty}$ (μM·h)	0.7
		Cl (mL/min/kg)	19.8
		Vd (L/kg)	1.1
	C57BL/6J mice (i.p. 10mg/kg)	$T_{1/2}$ (h)	1.5
		C_{max} (μM)	2.9
		$AUC_{0\sim\infty}$ (μM·h)	2.7
**Plasma Protein Binding**		% Unbound	1.4

When assessing the in vitro metabolic stability of LD4172, the indicated parameters represent the mean of three biologically independent samples. For the in vivo pharmacokinetic study, three mice were used for each route of administration, either intravenous (i.v.) or intraperitoneal (i.p.) injection.

Supplementary Figure 1

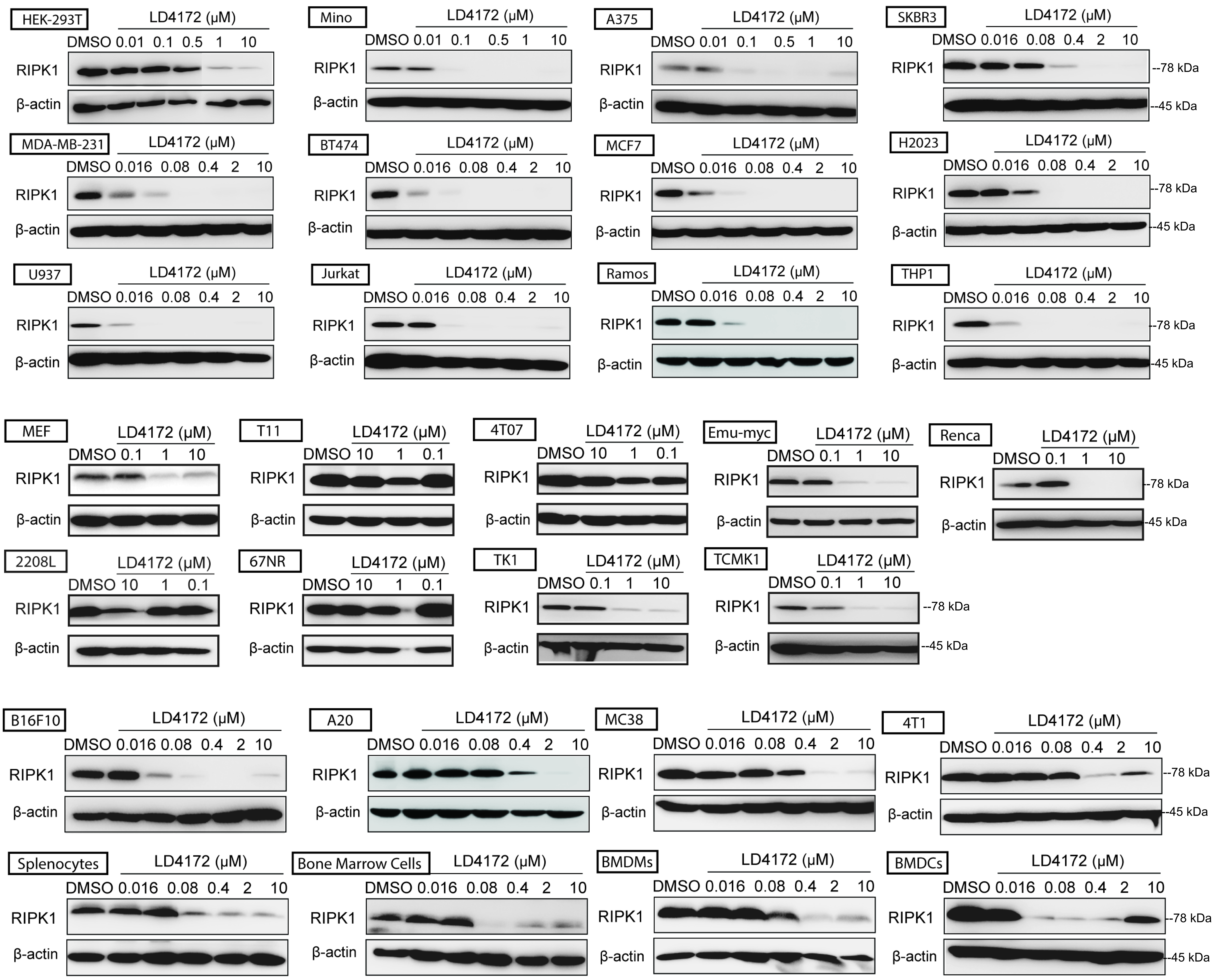

Degradation potency of LD4172 in a panel of cell lines. A Western blot was performed to assess RIPK1 levels in various cell lines treated with LD4172 at the indicated concentrations for 24 hours. Screening of LD4172 across different cell types was conducted in a single experiment. The degradation potency of LD4172 in each cell line was quantified using the DC50 value and Dmax, as shown in Fig. 2C.

Supplementary Figure 2

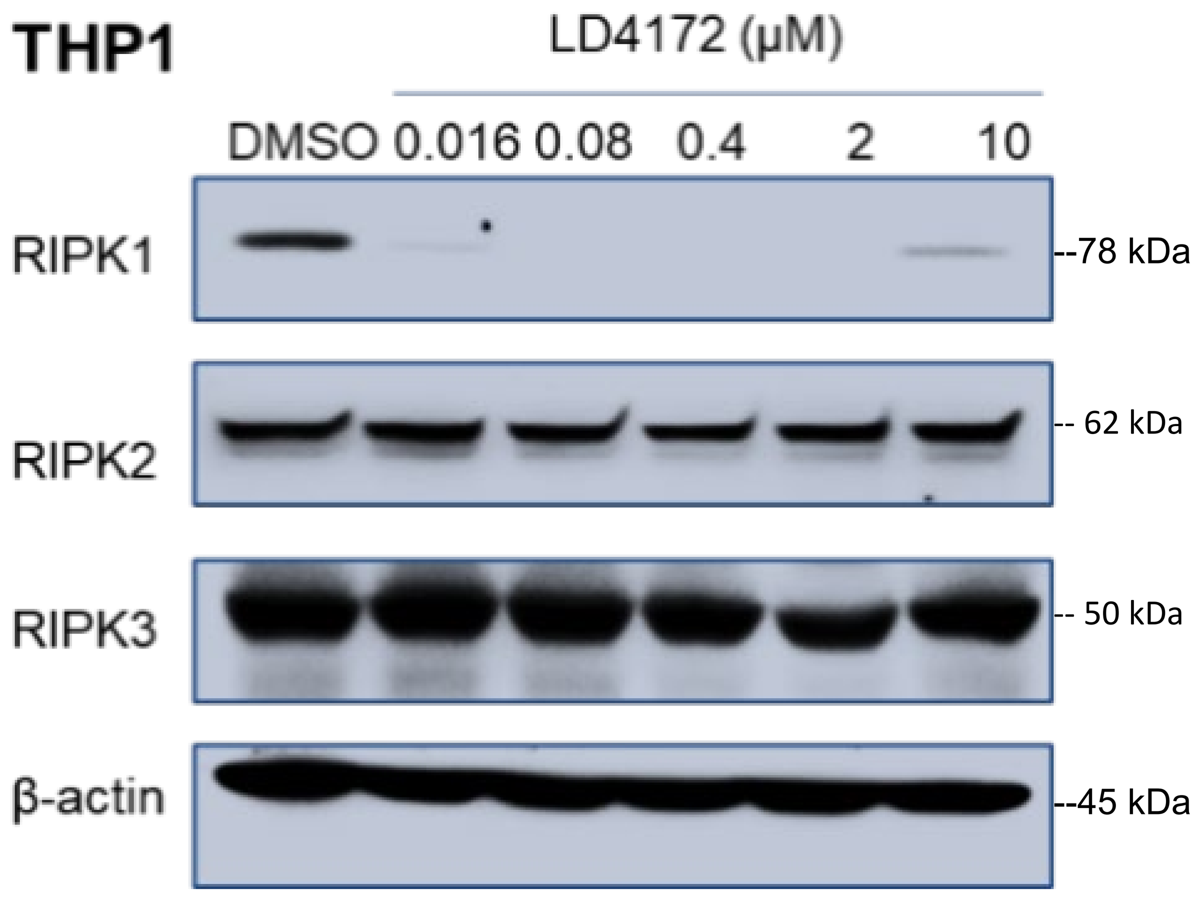

LD4172 showed high selectivity against RIPK1. Western blot analysis showing expression levels of RIPK1, RIPK2, and RIPK3 in THP1 cells following a 24-hour treatment with varying concentrations of LD4172. The experiments were conducted twice independently.

Supplementary Figure 3

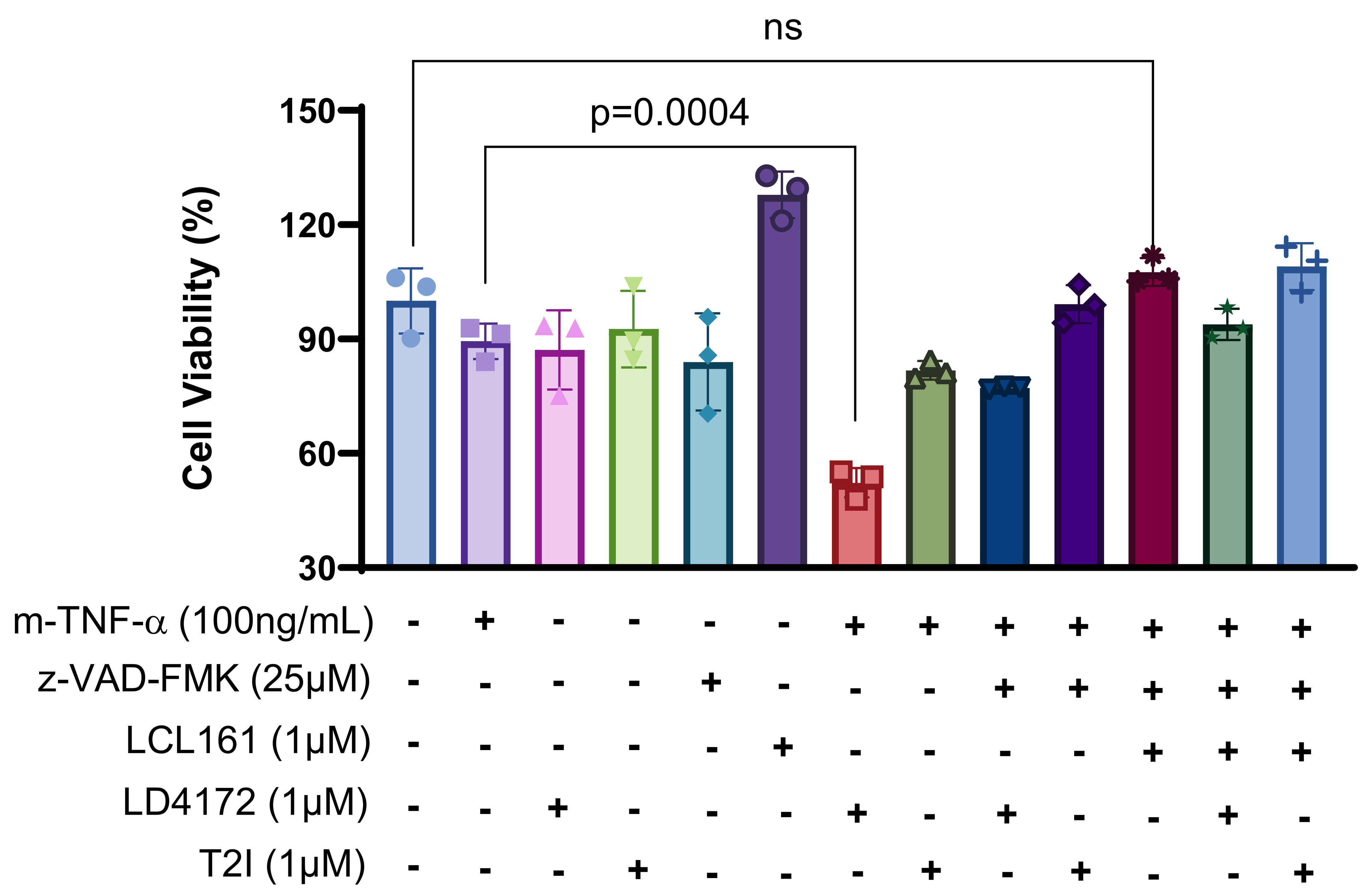

Cell viability of B16F10 cells with indicated treatment (72 h). Data represent the mean cell viability ± SD (n=3 biologically independent samples from three independent experiments). Statistical significance was determined using a two-tailed unpaired t-test, with P values indicated.

Supplementary Figure 4

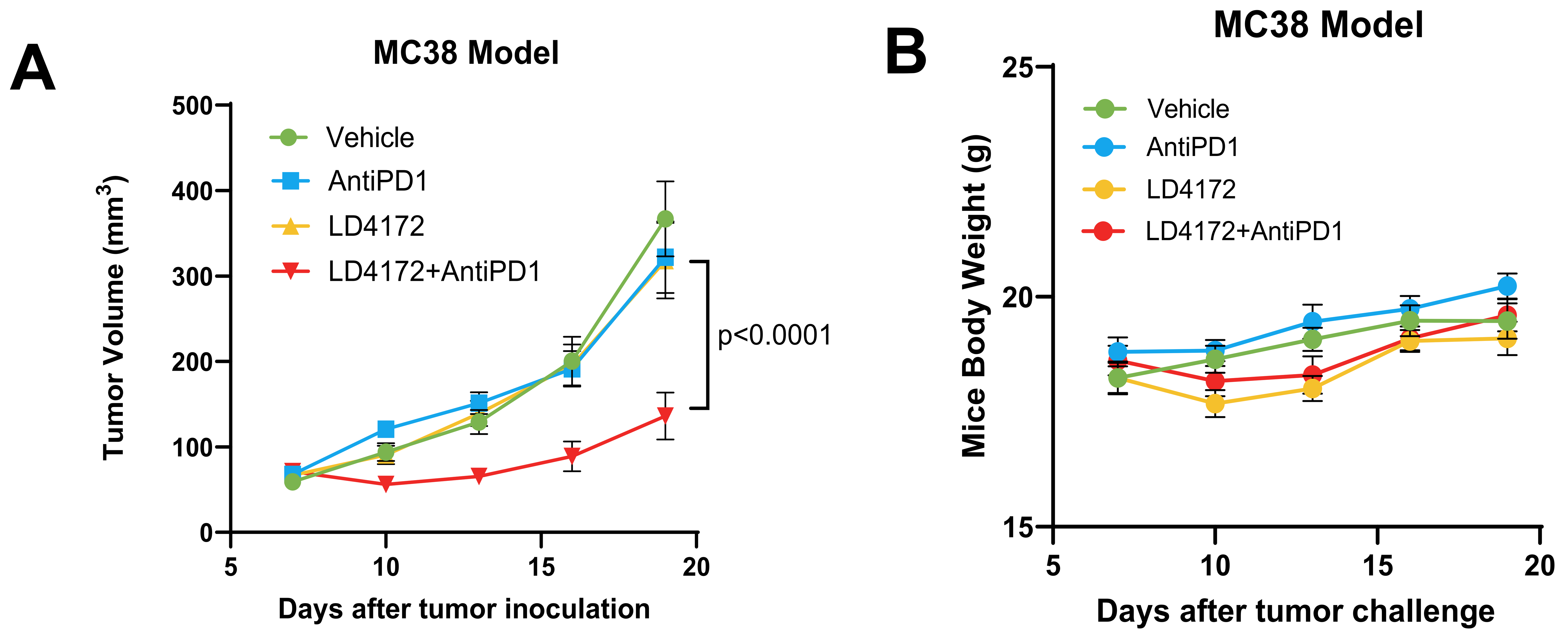

***In Vivo* efficacy of LD4172 in MC38 mouse model.** A. Tumor growth curve of mice bearing MC38 tumors treated with LD4172 and/or anti-PD1 (n=8 mice per group, one independent experiment). C57BL/6J mice were subcutaneously inoculated with 3 × 10^5 MC38 tumor cells. After seven days, when tumors reached approximately 80 mm^3, mice received either anti-PD1 (100 μg per dose, i.p.) every three days, LD4172 (20 mg/kg, i.p.) daily, a combination of LD4172 and anti-PD1 at the same doses, or the corresponding vehicle control. Each symbol represents the mean tumor volume, with error bars indicating SEM. Statistical analysis was conducted using two-way ANOVA followed by Sidak's multiple comparisons test, with significance levels indicated. B. Body weight curve of mice under the indicated treatments.

Supplementary Figure 5

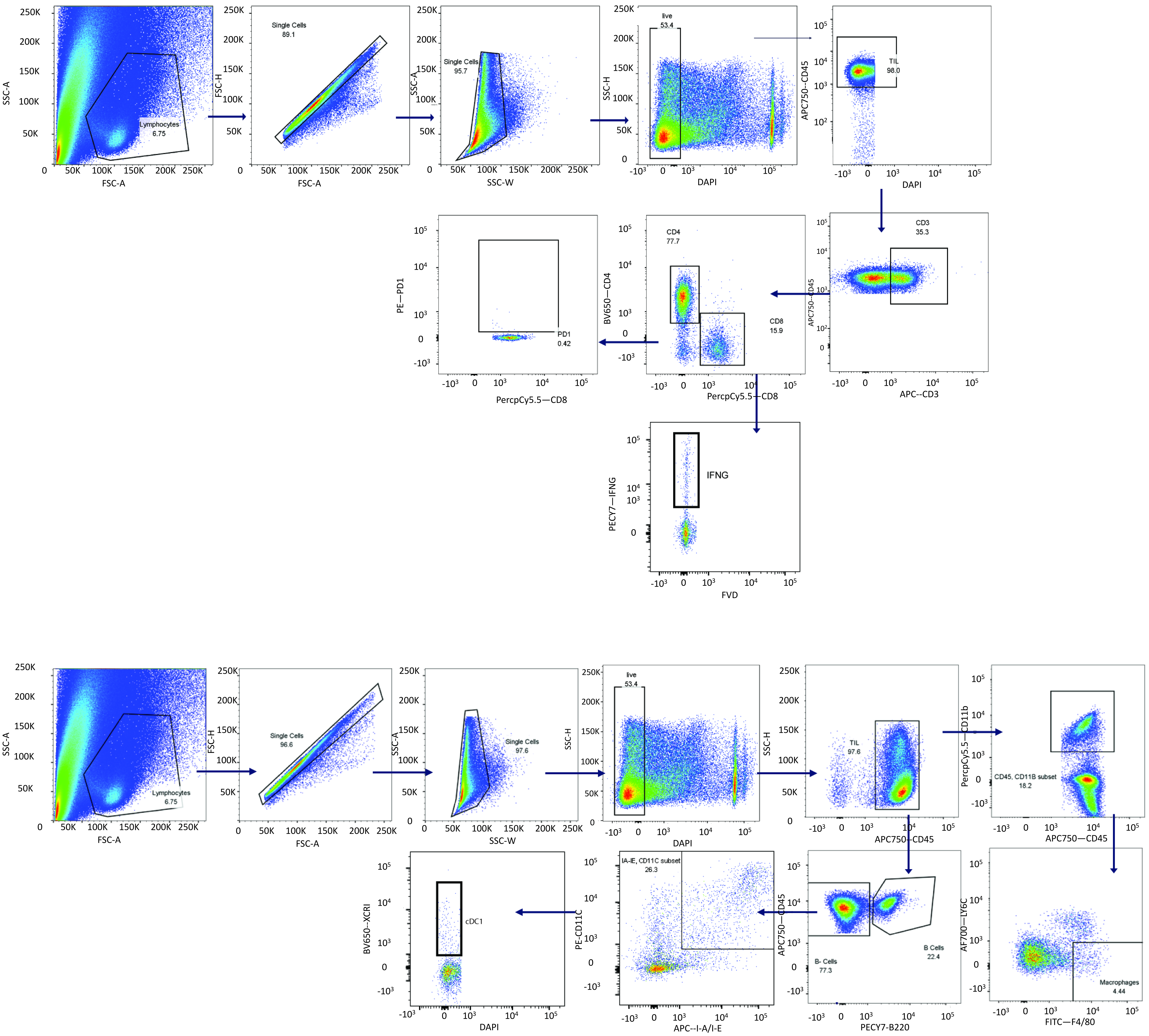

Representative plots showing the gating strategy for the data shown in Fig. 5.

Supplementary Figure 6

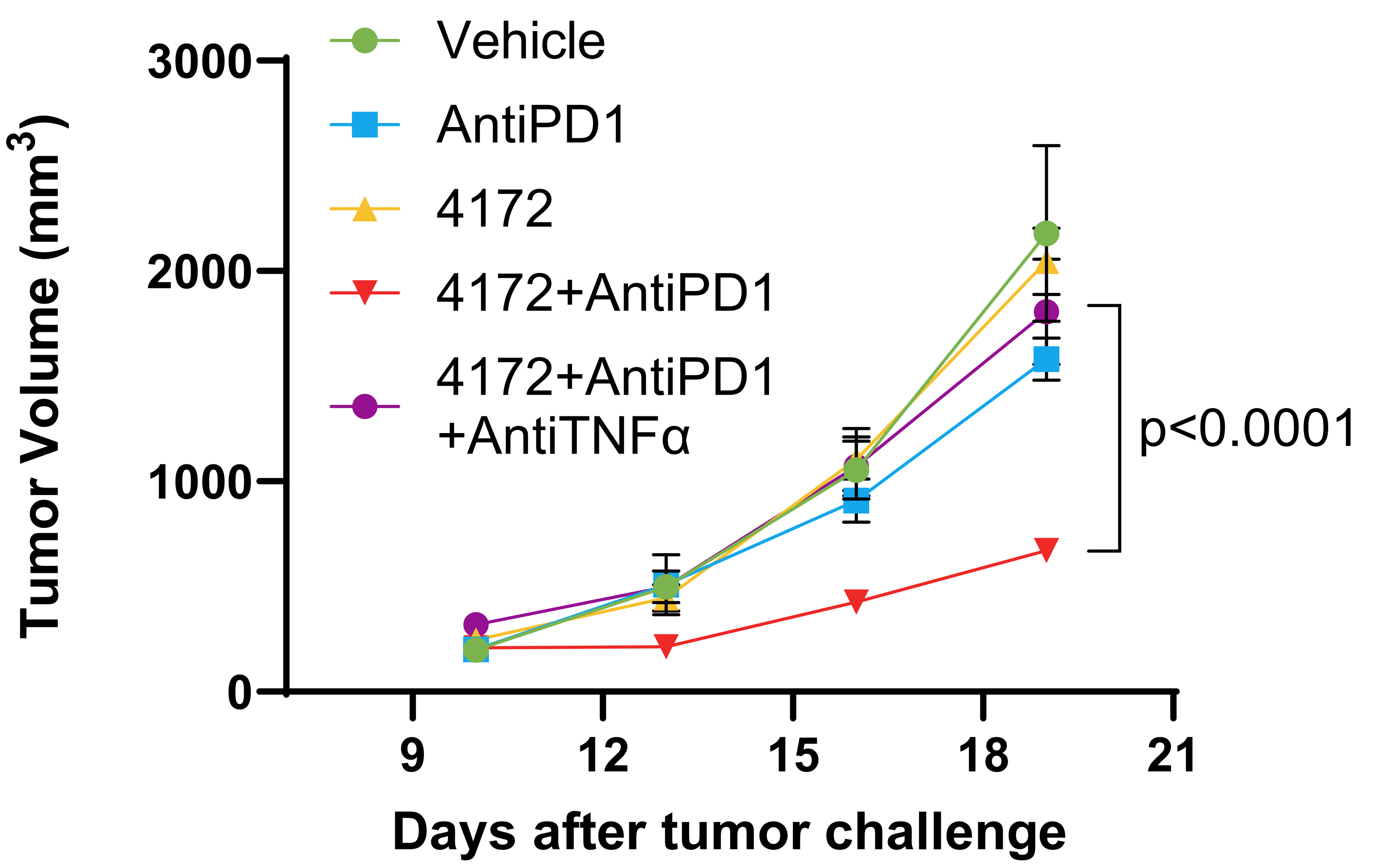

Anti-TNFα reversed the synergy between LD4172 and anti-PD1. C57B6/J mice were subcutaneously inoculated with 3×10^5 B16F10 tumor cells. After seven days (tumor size ~ 100 mm3), mice were treated every three days with anti-PD1 (100 µg per dose, i.p.), daily with LD4172 (20 mg/kg, i.p.), a combination of LD4172 and anti-PD1 (same dose as their individual doses), or combination plus anti-TNFα (200ug every three days,i.p.), or their corresponding vehicle control (n=5 mice/group, single experiment). Each symbol represents the mean tumor volume, with error bars indicating SEM. Statistical analysis was conducted using two-way ANOVA followed by Sidak's multiple comparisons test, with significance levels indicated.

Supplementary Figure 7

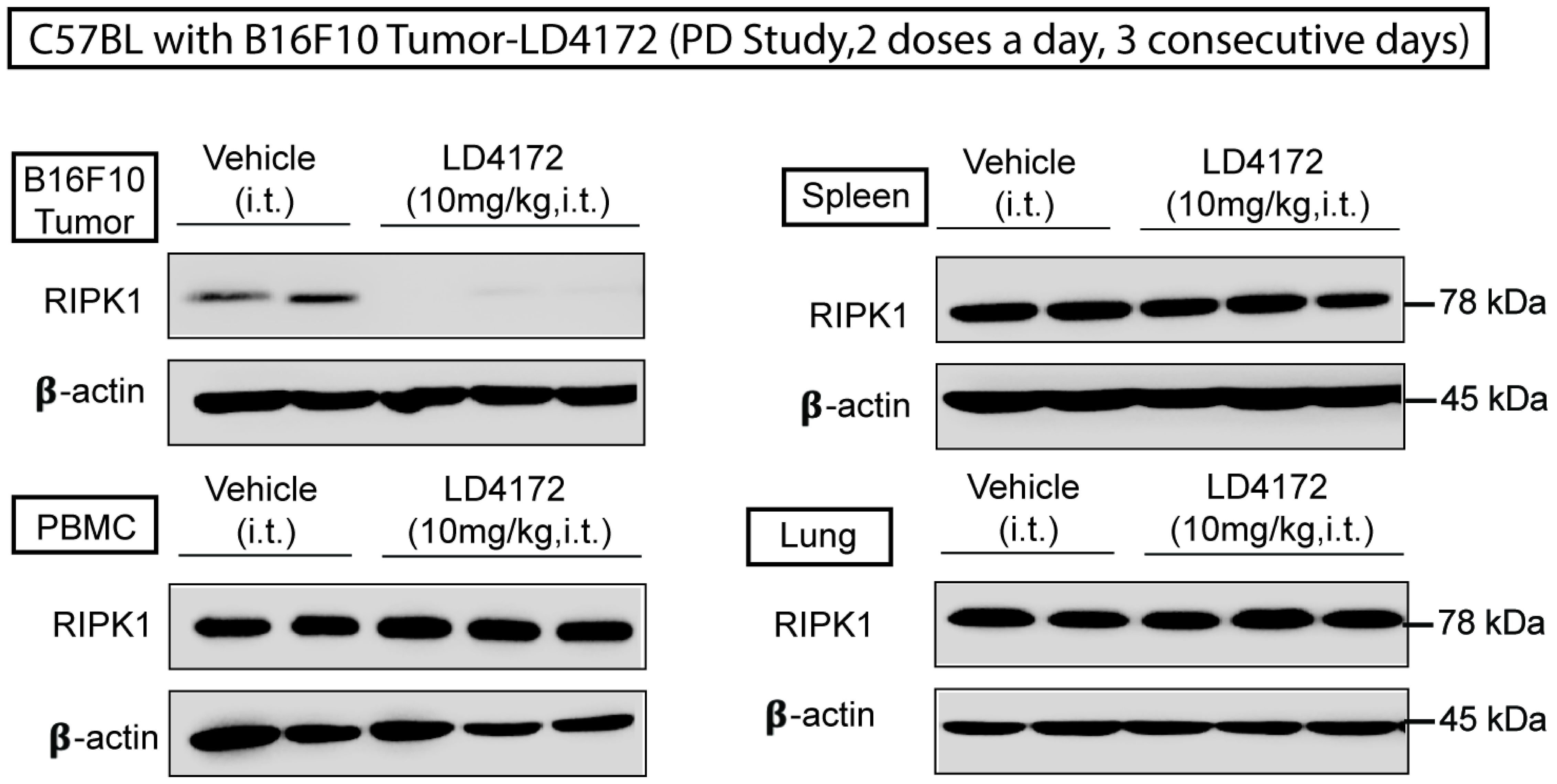

Pharmacodynamic (PD) properties of LD4172 with intratumoral administration. Representative immunoblots showing RIPK1 expression in various tissues of C57BL/6J mice treated with LD4172. Mice bearing syngeneic B16F10 tumors received intratumoral injections of LD4172 (10 mg/kg) twice daily for three days. Upon sacrifice, tissues were collected, and RIPK1 levels were quantified by Western blotting. Data are shown for two mice in the vehicle group and three mice in the LD4172 treatment group.

Supplementary Figure 8

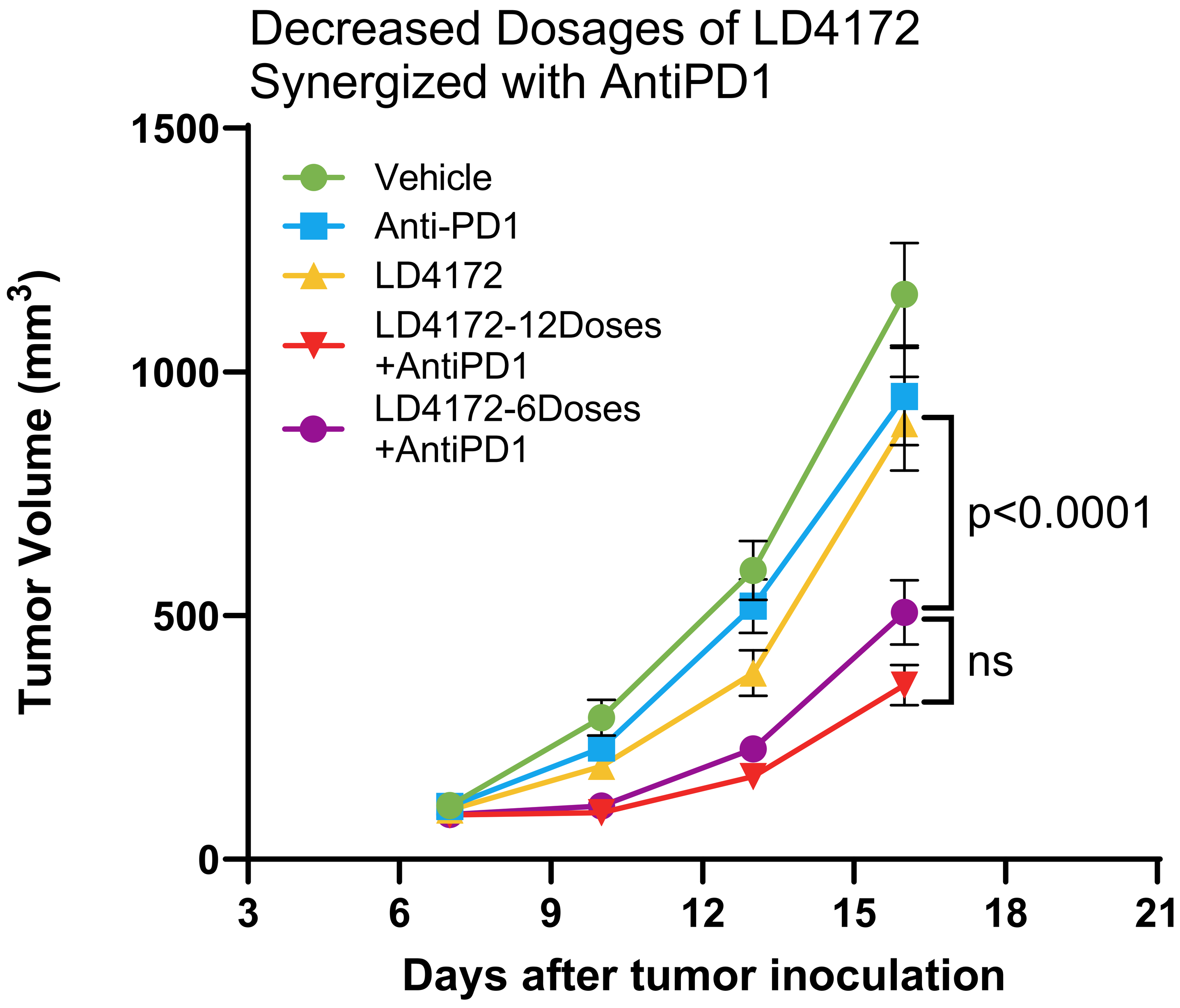

Tumor growth curve of mice with B16F10 tumors treated with reduced dosing frequency of LD4172. The administration of LD4172 was modified to a reduced dosage of 20 mg/kg every other day (n=8 mice/group, single experiment). Each symbol represents the mean tumor volume, with error bars indicating SEM. Statistical analysis was conducted using two-way ANOVA followed by Sidak's multiple comparisons test, with significance levels indicated.

Supplementary Figure 9

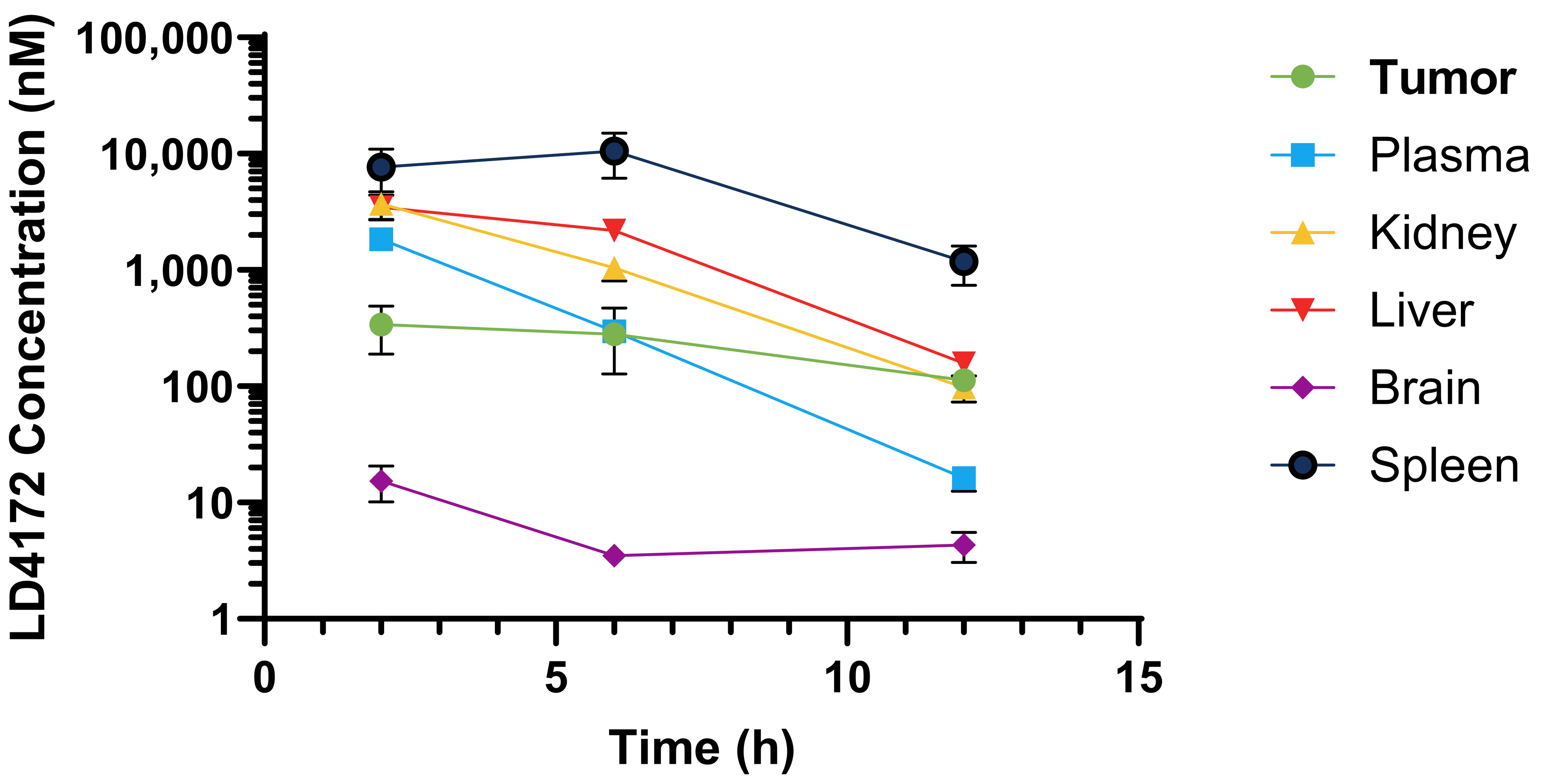

LD4172 concentrations in various tissues of B16F10 tumor-bearing C57BL/6J mice at different time points following a 20 mg/kg intraperitoneal (i.p.) administration (n=4 mice).

Supplementary Methods

All solvents and commercially available reagents were used as obtained. Oxygen and/or moisture sensitive reactions were carried out under a nitrogen atmosphere in oven- or flame-dried glassware. Chromatography purification was performed using a Teledyne ISCO Combiflash System Rf200 and/or Agilent 1260 Infinity Preparative LC System.

Nuclear magnetic resonance (NMR) analysis was performed on a Varian Palo Alto 400MHz NMR spectrometer. NMR chemical shifts are expressed in parts per million (ppm) and coupling constants (J) are expressed in Hz. Data were reported as follows: (s) singlet, (d) doublet, (t) triplet, (q) quartet, (m) multiplet, (br) broad.

Target compounds and/or intermediates were characterized by liquid chromatography/mass spectrometry (LCMS) using an Agilent 1260 Infinity LC/MS System. General conditions are as follows. Mass spectra were acquired on LC/MS systems using electrospray ionization methods from Agilent 1260 Infinity Analytic LC System with a SQ (single quadrupole) MS. $[M+H]^+$ refers to the protonated molecular ion of the chemical species. The mobile phase was 0.1% formic acid in water (solvent A) and 0.1% formic acid in ACN (solvent B).

Abbreviations used: H_2O for water, DCM for dichloromethane, DMSO for dimethyl sulfoxide, TEA for triethylamine, DIPEA for N,N-diisopropylethylamine, MeOH for methanol, DMF for N,Ndimethylformamide, HATU for 1-[bis(dimethylamino)methylene]-1H-1,2,3-triazolo[4,5-b]pyridinium 3-oxid hexafluoro-phosphate, TFA for trifluoroacetic acid, 2-Me-THF for 2-Methyltetrahydrofuran, ACN for acetonitrile, K_2CO_3 for potassium carbonate, RT for room temperature, N_2 for nitrogen.

Supplementary Figure 10

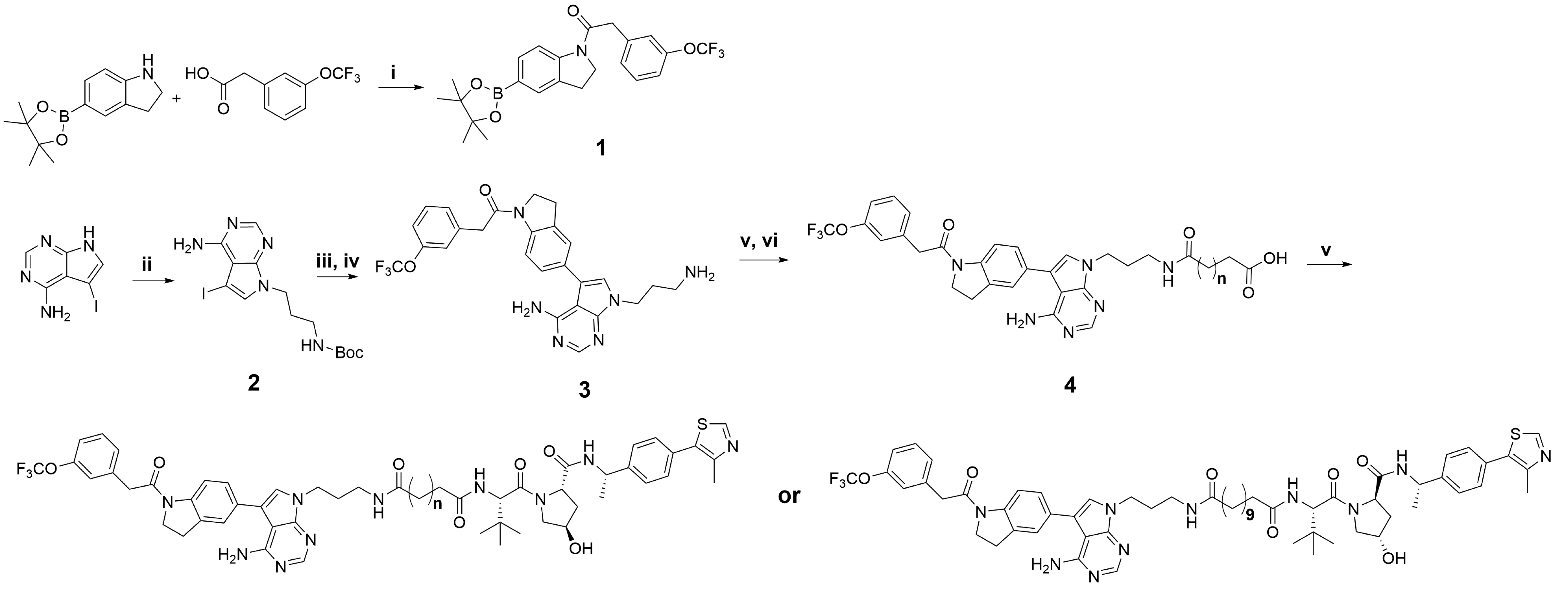

n = 1, 5-1; n = 3, 5-2;
n = 5, 5-3; n = 7, 5-4 (T2-VHL);
n = 9, 5-5 (LD4172); n = 11, 5-6

5-7 (LD4172-NC)

Synthesis of type II warhead based PROTACs

Reagents and conditions: i) HATU, TEA, DMF, 65°C; ii) tert-butyl (3-bromopropyl)carbamate, K_2CO_3, DMF, 50°C; iii) $Pd(dppf)_2Cl_2.CH_2Cl_2$, K_2CO_3, dioxane/H2O = 5/1, 90 °C; iv) 3 N HCl in MeOH, DCM, RT; v) Various carboxylate acid, HATU, DIPEA, DMF, RT; vi) TFA, DCM, RT; vii) (S,R,S)-AHPC-Me hydrochlorideor or (S,S,R)-AHPC-Me hydrochloride, HATU, TEA, DMF, RT.

Supplementary Figure 11

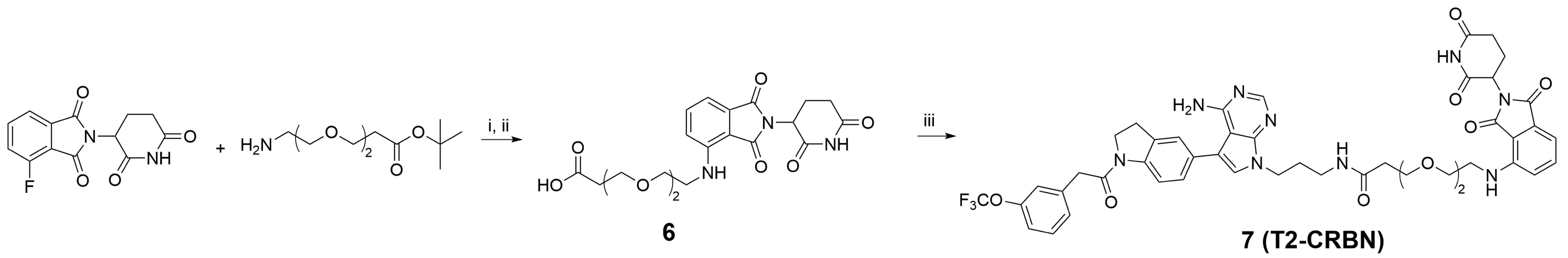

Synthesis of type II warhead based PROTACs

Reagents and conditions: i) DIPEA, DMF, 90°C; ii) TFA, DCM, RT; iii) 3, HATU, DIPEA, DMF, RT.

Supplementary Figure 12

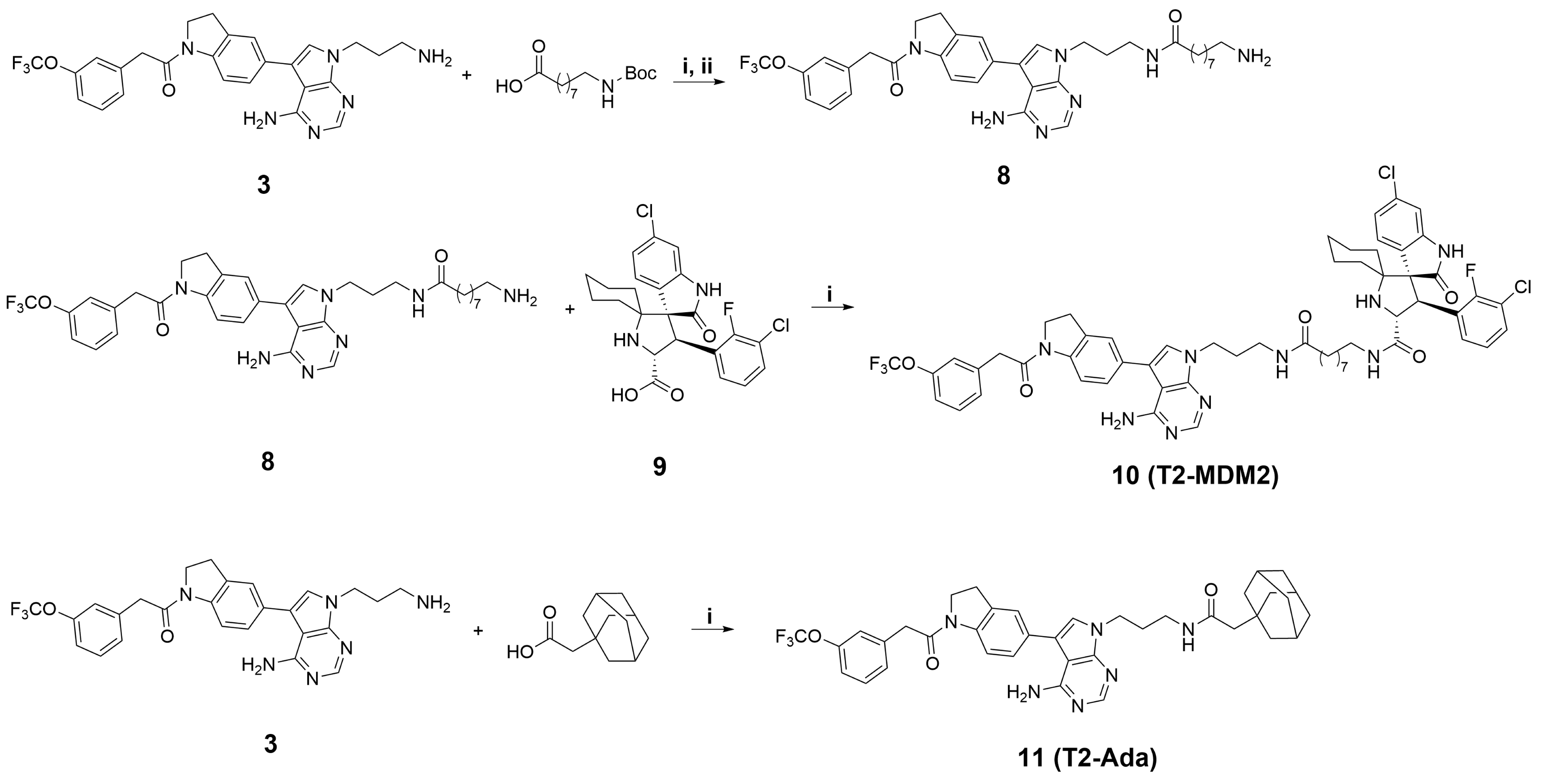

Synthesis of type II warhead based PROTACs

Reagents and conditions: i) HATU, DIPEA, DMF, RT; ii) TFA, DCM, RT.

Supplementary Figure 13

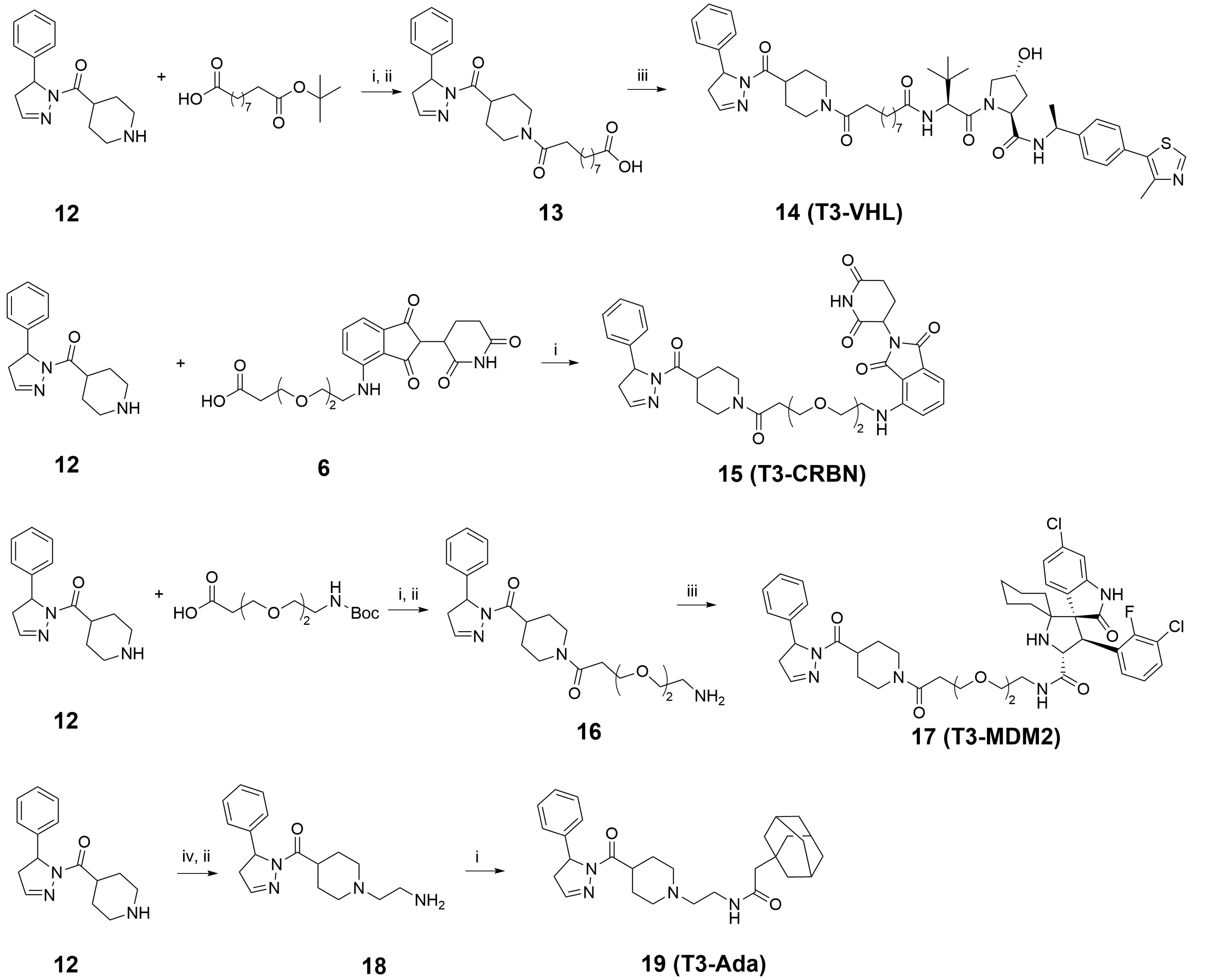

Synthesis of type III warhead based PROTACs

Reagents and conditions: i) HATU, DIPEA, 2-Me-THF, RT; ii) TFA, DCM, RT; iii) (S,R,S)-AHPC-Me hydrochlorideor or 9 or 2-(adamantan-1-yl)acetic acid, HATU, DIPEA, DMF, RT; iv) tert-butyl (2-bromoethyl)carbamate, K_2CO_3, DMF, RT.

Supplementary Figure 14

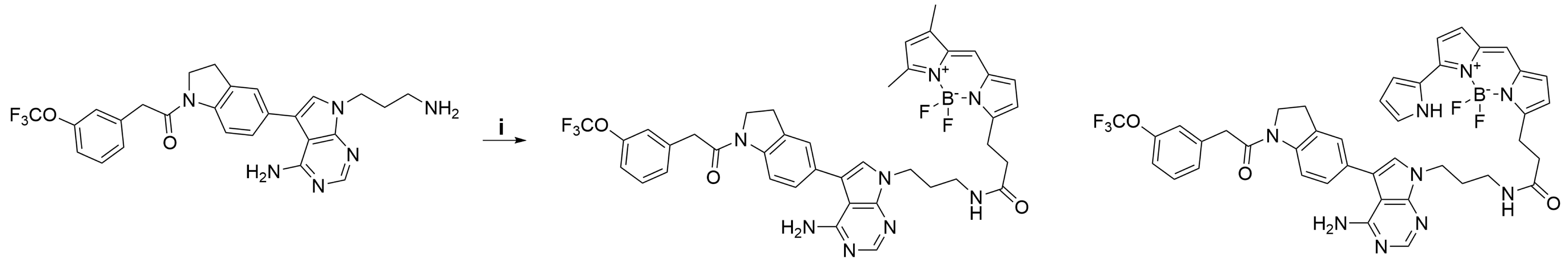

3 T2-488 T2-590

Synthesis of RIPK1 tracers

Reagents and conditions: i) BDP FL NHS ester or BDP 576/589 NHS ester, DIPEA, DMF, RT

Synthetic procedure

1-(5-(4,4,5,5-tetramethyl-1,3,2-dioxaborolan-2-yl)indolin-1-yl)-2-(3-(trifluoromethoxy)phenyl)ethan-1-one (1): To a solution of 5-(4,4,5,5-tetramethyl-1,3,2-dioxaborolan-2-yl)indoline (1.5 g, 6.12 mmol) in DMF (50 mL) was added 2-(3-(trifluoromethoxy)phenyl)acetic acid (2.81 g, 6.7 mmol) and TEA (1.77 mL, 12.24 mmol). The mixture was stirred at 65 °C for 3 hours before adding water (100 mL). The resulting mixture was extracted with ethyl acetate (2×100 mL), the combined organic phases were dried over anhydrous Na_2SO_4, and concentrated under reduced pressure to give the title compound without further purification (2.18 g, 80% yield). ^{1}H NMR (400 MHz, $CDCl_3$) δ 8.21 (d, *J* = 8.1 Hz, 1H), 7.67 (d, *J* = 8.2 Hz, 1H), 7.62 (s, 1H), 7.37 (t, *J* = 7.9 Hz, 1H), 7.24 (s, 1H), 7.17 – 7.10 (m, 2H), 4.09 (t, *J* = 8.5 Hz, 2H), 3.81 (s, 2H), 3.18 (t, *J* = 8.4 Hz, 2H), 1.33 (s, 12H). ^{13}C NMR (100 MHz, $CDCl_3$) δ 168.38, 149.37, 145.38, 136.2, 134.93,130.85, 130.39, 129.92, 127.72, 121.96, 119.42, 116.40, 83.69, 48.29, 42.84, 27.71, 24.84. MS (ESI) m/z: $[M + H]^+$, 448.2

tert-butyl (3-(4-amino-5-iodo-7H-pyrrolo[2,3-d]pyrimidin-7-yl)propyl)carbamate (2): To a solution of 5-iodo-7H-pyrrolo[2,3-d]pyrimidin-4-amine (1.0 g, 3.8 mmol) and tert-butyl (3-bromopropyl)carbamate (1.1 g, 4.6 mmol) in DMF (40 mL) was added K_2CO_3 (0.63 g, 4.6 mmol). The mixture was stirred at 50 °C for 5 hours before adding water (100 mL). The resulting mixture was extracted with DCM (2×100 mL) and the combined organic phases were dried over anhydrous Na_2SO_4 and concentrated under reduced pressure to give the title compound without further purification (1.15 g, 72% yield). ^{1}H NMR (400 MHz, DMSO-d_6) δ 8.06 (s, 1H), 7.46 (s, 1H), 6.85 (s, 1H), 6.57 (s, 2H), 4.07 (t, *J* = 6.8 Hz, 2H), 2.84 (dd, *J* = 12.2, 6.1 Hz, 2H), 1.89 – 1.71 (m, 2H), 1.35 (s, 9H). MS (ESI) m/z: $[M + H]^+$, 418.1

1-(5-(4-amino-7-(3-aminopropyl)-7H-pyrrolo[2,3-d]pyrimidin-5-yl)indolin-1-yl)-2-(3-(trifluoromethoxy)phenyl)ethan-1-one (3): A solution of **1** (1.0 g, 2.2 mmol), **2** (766 mg, 1.83 mmol), K_2CO_3 (505 mg, 3.66 mmol) and $Pd(dppf)_2Cl_2.CH_2Cl_2$ (75 mg, 0.09 mmol) in 1,4-dioxane (50 mL) and water (10 mL) was degassed and fulfilled with N_2 for three times. Then the mixture was stirred at 90 °C for 12 hours before it was quenched with water. The resulting mixture was extracted with DCM (3×100 mL), the combined organic phases were dried over anhydrous Na_2SO_4 and concentrated under reduced pressure. The residue was purified by silica gel flash column chromatography to give the product. The product was dissolved in DCM (30 mL) and the solution was slowly added 3 N HCl in methanol (2 mL) in an ice bath. Then the solution was stirred at RT overnight. The mixture was filtered and the solid was dried under vacuum to give the title compound (330 mg, 36% yield, two steps). ^{1}H NMR (400 MHz, DMSO-d_6) δ 8.49 (s, 1H), 8.17 – 8.01 (m, 4H), 7.69 (s, 1H), 7.46 (t, *J* = 7.9 Hz, 1H), 7.34 – 7.28 (m, 3H), 7.24 (d, *J* = 8.2 Hz, 3H), 4.34 (t, *J* = 6.6 Hz, 2H), 4.24 (t, *J* = 8.5 Hz, 2H), 3.97 (s, 2H), 3.22 (d, *J* = 8.2 Hz, 2H), 2.76 (d, *J* = 6.5 Hz, 2H), 2.16 – 2.05 (m, 2H). MS (ESI) m/z: $[M + H]^+$, 511.2

General Procedure for Synthesis of Compounds 5: To a solution of **3** (50 mg, 0.1 mmol) and carboxylic acid (0.12 mmol) in DMF (5 mL) was added HATU (57 mg, 0.15 mmol) and DIPEA (43 μL, 0.3 mmol). The mixture was stirred at RT overnight. The reaction was concentrated and then was purified by silica gel flash column chromatography to give the title compound. The compound was dissolved in DCM (2 mL) and was added TFA (0.5 mL). The mixture was stirred at RT for 5 hours before it was concentrated under reduced pressure. The crude product was used directly in the next step.

To a solution of **4** (0.04 mmol) and (S,R,S)-AHPC-Me hydrochloride or (S,S,R)-AHPC-Me hydrochloride (0.05 mmol) in DMF (5 mL) was added HATU (28 mg, 0.07 mmol) and DIPEA (17 μL, 0.12 mmol). The mixture was stirred at RT for 1 hour. The reaction was concentrated and then was purified by reverse phase preparative HPLC to provide compound **5**.

N1-(3-(4-amino-5-(1-(2-(3-(trifluoromethoxy)phenyl)acetyl)indolin-5-yl)-7H-pyrrolo[2,3-d]pyrimidin-7-yl)propyl)-N4-((S)-1-((2S,4R)-4-hydroxy-2-(((S)-1-(4-(4-methylthiazol-5-yl)phenyl)ethyl)carbamoyl)pyrrolidin-1-yl)-3,3-dimethyl-1-oxobutan-2-yl)succinimide (5-1, 63% yield*)*: ^{1}H NMR (400 MHz, DMSO-d_6) δ 8.97 (s, 1H), 8.49 – 8.32 (m, 1H), 8.24 – 7.92 (m, 1H), 7.84 (s, 2H), 7.41 (s, 3H), 7.37 (s, 2H), 7.25 (d, *J* = 12.1 Hz, 3H), 7.12 – 6.85 (m, 1H), 5.08 (s, 1H), 4.90 (s, 1H), 4.43 (d, *J* = 23.0 Hz, 1H), 4.21 (d, *J* = 36.1 Hz, 2H), 3.91 (s, 1H), 3.57 (s, 2H), 2.49 (s, 4H), 2.44 (s, 3H), 2.35 (s, 1H), 2.29 (s, 2H), 1.99 (s, 1H), 1.76 (s, 2H), 1.36 (s, 3H), 0.90 (s, 9H). MS (ESI) m/z: [M + H]$^+$, 1037.4

N1-(3-(4-amino-5-(1-(2-(3-(trifluoromethoxy)phenyl)acetyl)indolin-5-yl)-7H-pyrrolo[2,3-d]pyrimidin-7-yl)propyl)-N6-((S)-1-((2S,4R)-4-hydroxy-2-(((S)-1-(4-(4-methylthiazol-5-yl)phenyl)ethyl)carbamoyl)pyrrolidin-1-yl)-3,3-dimethyl-1-oxobutan-2-yl)adipamide (5-2, 65% yield*)*: ^{1}H NMR (400 MHz, DMSO-d_6) δ 8.97 (s, 1H), 8.62 (s, 2H), 8.41 (d, *J* = 8.4 Hz, 1H), 8.36 (d, *J* = 8.0 Hz, 1H), 8.10 (d, *J* = 11.6 Hz, 1H), 7.85 (s, 1H), 7.77 (d, *J* = 8.9 Hz, 1H), 7.43 (d, *J* = 22.0 Hz, 3H), 7.37 (s, 1H), 7.31 (s, 2H), 7.27 – 7.20 (m, 1H), 5.09 (s, 1H), 4.89 (s, 1H), 4.48 (d, *J* = 9.5 Hz, 1H), 4.40 (s, 1H), 4.24 (d, *J* = 8.8 Hz, 2H), 4.14 (s, 2H), 3.95 (s, 2H), 3.58 (s, 2H), 3.10 (d, *J* = 6.2 Hz, 3H), 2.44 (s, 3H), 2.21 (s, 2H), 2.05 (s, 2H), 1.89 (s, 2H), 1.45 (s, 2H), 1.35 (d, *J* = 6.9 Hz, 2H), 1.23 (s, 8H), 0.89 (s, 8H). MS (ESI) m/z: [M + H]$^+$, 1065.5

N1-(3-(4-amino-5-(1-(2-(3-(trifluoromethoxy)phenyl)acetyl)indolin-5-yl)-7H-pyrrolo[2,3-d]pyrimidin-7-yl)propyl)-N8-((S)-1-((2S,4R)-4-hydroxy-2-(((S)-1-(4-(4-methylthiazol-5-yl)phenyl)ethyl)carbamoyl)pyrrolidin-1-yl)-3,3-dimethyl-1-oxobutan-2-yl)octanediamide (5-3, 49% yield*)*: ^{1}H NMR (400 MHz, DMSO-d_6) δ 8.97 (s, 1H), 8.36 (d, *J* = 6.9 Hz, 1H), 8.25 – 7.89 (m, 1H), 7.76 (d, *J* = 7.0 Hz, 2H), 7.42 (d, *J* = 7.2 Hz, 3H), 7.36 (d, *J* = 7.2 Hz, 2H), 7.31 – 7.15 (m, 4H), 5.09 (s, 1H), 4.96 – 4.84 (m, 1H), 4.50 (d, *J* = 9.5 Hz, 1H), 4.41 (t, *J* = 7.7 Hz, 1H), 4.21 (d, *J* = 38.5 Hz, 2H), 3.94 (d, *J* = 22.2 Hz, 1H), 3.58 (s, 2H), 2.44 (s, 3H), 2.14 (d, *J* = 45.0 Hz, 2H), 1.99 (s, 2H), 1.77 (s, 2H), 1.43 (s, 4H), 1.35 (d, *J* = 6.3 Hz, 3H), 1.20 (s, 5H), 0.91 (s, 9H). MS (ESI) m/z: [M + H]$^+$, 1093.5

N1-(3-(4-amino-5-(1-(2-(3-(trifluoromethoxy)phenyl)acetyl)indolin-5-yl)-7H-pyrrolo[2,3-d]pyrimidin-7-yl)propyl)-N10-((S)-1-((2S,4R)-4-hydroxy-2-(((S)-1-(4-(4-methylthiazol-5-yl)phenyl)ethyl)carbamoyl)pyrrolidin-1-yl)-3,3-dimethyl-1-oxobutan-2-yl)decanediamide (5-4, T2-VHL, 51% yield*)*: 1H NMR (400 MHz, DMSO-d_6) δ 8.96 (s, 1H), 8.36 (d, *J* = 7.3 Hz, 1H), 8.27 (s, 1H), 8.10 (d, *J* = 9.4 Hz, 2H), 7.83 (s, 1H), 7.77 (d, J = 8.9 Hz, 1H), 7.43 (dd, *J* = 14.3, 8.2 Hz, 4H), 7.36 (d, *J* = 8.2 Hz, 3H), 7.30 (s, 3H), 7.28 – 7.19 (m, 2H), 6.00 (s, 1H), 4.89 (s, 1H), 4.49 (d, *J* = 9.1 Hz, 1H), 4.40 (s, 1H), 4.24 (d, *J* = 9.2 Hz, 4H), 4.13 (s, 2H), 3.95 (s, 2H), 3.58 (s, 4H), 3.23 (d, *J* = 7.8 Hz, 4H), 3.02 (s, 3H), 2.43 (s, 3H), 2.20 (s, 1H), 2.03 (s, 5H), 1.87 (s, 2H), 1.77 (s, 1H), 1.35 (d, *J* = 6.9 Hz, 4H), 1.21 (s, 12H), 0.91 (s, 9H). ^{13}C NMR (100 MHz, DMSO-d_6) δ 172.54, 171.07, 170.06, 157.67, 151.92, 150.50, 148.21, 145.11, 142.23, 138.47, 130.42, 129.37, 127.60, 126.83, 125.39, 123.69, 122.70, 119.46, 116.63, 69.21, 56.79, 48.15, 41.70, 38.19, 36.39, 35.96, 35.64, 35.35, 30.37, 29.17, 27.96, 26.90, 25.81, 22.89, 16.44. MS (ESI) m/z: [M + H]$^+$, 1121.5

N1-(3-(4-amino-5-(1-(2-(3-(trifluoromethoxy)phenyl)acetyl)indolin-5-yl)-7H-pyrrolo[2,3-d]pyrimidin-7-yl)propyl)-N4-((S)-1-((2S,4R)-4-hydroxy-2-(((S)-1-(4-(4-methylthiazol-5-yl)phenyl)ethyl)carbamoyl)pyrrolidin-1-yl)-3,3-dimethyl-1-oxobutan-2-yl)succinimide (5-5, LD4172, 56% yield*):* ^{1}H NMR (400 MHz, DMSO-d_6) δ 8.96 (s, 1H), 8.36 (d, J = 7.8 Hz, 1H), 8.11 (d, J = 8.3 Hz, 1H), 8.07 (d, J = 8.8 Hz, 1H), 7.77 (d, J = 9.4 Hz, 1H), 7.67 (t, J = 5.5 Hz, 1H), 7.46 (t, J = 7.9 Hz, 1H), 7.41 (d, J = 8.3 Hz, 2H), 7.38 – 7.31 (m, 4H), 7.29 (t, J = 6.6 Hz, 2H), 7.25 (d, J = 7.6 Hz, 2H), 6.98 (s, 2H), 6.52 (s, 1H), 4.90 (dd, J = 14.1, 6.8 Hz, 1H), 4.49 (d, J = 9.3 Hz, 1H), 4.39 (t, J = 8.0 Hz, 1H), 4.25 (d, J = 7.5 Hz, 2H), 4.14 (t, J = 7.3 Hz, 2H), 3.96 (s, 2H), 3.57 (s, 1H), 3.22 (d, J = 8.4 Hz, 2H), 2.87 – 2.79 (m, 2H), 2.43 (s, 3H), 2.20 (dd, J = 14.3, 7.2 Hz, 1H), 2.07 (dd, J = 13.7, 7.0 Hz, 1H), 2.02 – 1.94 (m, 1H), 1.92 (t, J = 7.4 Hz, 2H), 1.76 (ddd, J = 12.8, 8.4, 4.6 Hz, 1H), 1.69 – 1.58 (m, 2H), 1.34 (d, J = 6.9 Hz, 3H), 1.18 (d, J = 19.8 Hz, 16H), 0.90 (s, 9H). ^{13}C NMR (100 MHz, DMSO-d_6) δ 172.40, 171.04, 170.03, 169.15, 157.51, 151.91, 151.39, 148.67, 148.18, 145.10, 143.27, 138.34, 136.67, 133.03, 131.54, 130.46, 130.11, 129.45, 129.25, 128.08, 126.80, 126.55 (q, J = 206 Hz), 122.68, 119.46, 116.39, 102.89, 99.08, 69.18, 58.96, 56.75, 48.34, 48.13, 41.70, 38.17, 36.30, 35.88, 35.62, 35.32, 30.38, 29.25, 27.85, 26.86, 25.87, 25.64, 22.88, 16.43. MS (ESI) m/z: $[M + H]^+$, 1149.5

N1-(3-(4-amino-5-(1-(2-(3-(trifluoromethoxy)phenyl)acetyl)indolin-5-yl)-7H-pyrrolo[2,3-d]pyrimidin-7-yl)propyl)-N14-((S)-1-((2S,4R)-4-hydroxy-2-(((S)-1-(4-(4-methylthiazol-5-yl)phenyl)ethyl)carbamoyl)pyrrolidin-1-yl)-3,3-dimethyl-1-oxobutan-2-yl)tetradecanediamide (5-6, 53% yield): ^{1}H NMR (400 MHz, DMSO-d_6) δ 8.97 (s, 1H), 8.36 (d, J = 7.6 Hz, 1H), 8.25 (s, 2H), 8.10 (d, J = 8.7 Hz, 2H), 7.83 (s, 1H), 7.76 (d, J = 9.5 Hz, 1H), 7.43 (dd, J = 15.5, 7.4 Hz, 3H), 7.39 – 7.32 (m, 3H), 7.30 (s, 3H), 7.27 – 7.17 (m, 2H), 6.05 (s, 1H), 4.90 (s, 1H), 4.50 (d, J = 9.1 Hz, 1H), 4.39 (d, J = 8.3 Hz, 1H), 4.28 – 4.19 (m, 4H), 4.14 (s, 2H), 3.96 (s, 2H), 3.22 (s, 2H), 3.02 (s, 2H), 2.44 (s, 3H), 2.21 (s, 1H), 2.13 – 1.95 (m, 4H), 1.88 (s, 2H), 1.77 (s, 1H), 1.35 (d, J = 6.4 Hz, 3H), 1.20 (s, 20H), 0.91 (s, 9H). MS (ESI) m/z: $[M + H]^+$, 1177.6

N1-(3-(4-amino-5-(1-(2-(3-(trifluoromethoxy)phenyl)acetyl)indolin-5-yl)-7H-pyrrolo[2,3-d]pyrimidin-7-yl)propyl)-N12-((S)-1-((2R,4S)-4-hydroxy-2-(((S)-1-(4-(4-methylthiazol-5-yl)phenyl)ethyl)carbamoyl)pyrrolidin-1-yl)-3,3-dimethyl-1-oxobutan-2-yl)dodecanediamide (5-7, LD4172-NC, 35% yield). ^{1}H NMR (400 MHz, DMSO-d_6) δ 8.95 (s, 1H), 8.14 (s, 1H), 8.09 (d, J = 9.5 Hz, 2H), 8.00 (d, J = 8.0 Hz, 1H), 7.93 (s, 1H), 7.88 (d, J = 7.6 Hz, 1H), 7.81 (d, J = 5.6 Hz, 1H), 7.46 (dd, J = 14.7, 7.0 Hz, 1H), 7.39 (d, J = 9.1 Hz, 3H), 7.31 (d, J = 13.4 Hz, 4H), 7.22 (dd, J = 14.7, 8.6 Hz, 2H), 4.93 – 4.82 (m, 1H), 4.36 (t, J = 6.6 Hz, 2H), 4.21 (t, J = 8.4 Hz, 2H), 4.12 (t, J = 6.7 Hz, 2H), 3.94 (s, 2H), 3.79 (dd, J = 10.3, 5.4 Hz, 1H), 3.47 (dd, J = 10.2, 3.8 Hz, 2H), 3.21 (t, J = 8.3 Hz, 2H), 3.02 – 2.98 (m, 2H), 2.40 (d, J = 13.1 Hz, 3H), 2.27 – 2.13 (m, 1H), 2.10 – 1.98 (m, 4H), 1.97 – 1.93 (m, 1H), 1.90 – 1.84 (m, 2H), 1.28 (d, J = 6.9 Hz, 3H), 1.27 – 1.06 (m, 16H), 0.93 (s, 9H). ^{13}C NMR (100 MHz, DMSO-d_6) δ 172.59, 168.89, 162.73, 157.64, 151.88, 150.47, 138.43, 133.25, 130.35, 130.07, 129.43, 129.19, 128.20 (q, J = 206 Hz),127.57, 126.97, 125.35, 123.64, 122.67, 119.42, 116.61, 115.44, 100.38, 68.80, 59.13, 57.54, 55.61, 48.29, 47.92, 42.07, 41.67, 36.28, 35.93, 35.16, 34.65, 31.21, 30.35, 29.86, 28.71, 27.94, 27.08, 26.48, 26.01, 25.57, 22.94, 16.42. MS (ESI) m/z: $[M + H]^+$, 1149.5

3-(2-(2-((2-(2,6-dioxopiperidin-3-yl)-1,3-dioxoisoindolin-4-yl)amino)ethoxy)ethoxy)propanoic acid (6): To a solution of 3-(4-fluoro-1,3-dioxo-2,3-dihydro-1H-inden-2-yl)piperidine-2,6-dione (50 mg, 0.18 mmol) in DMF (10 mL) was added tert-butyl 3-(2-(2-aminoethoxy)ethoxy)propanoate (50 mg, 0.22 mmol) and DIPEA (51 μL, 0.36 mmol). The mixture was stirred at 90 °C for 2 hours. The reaction was concentrated and then was purified by silica gel flash column chromatography to give the title compound. The compound was dissolved in DCM (2 mL) and TFA (0.5 mL). The mixture was stirred at RT for 7 hours before it was concentrated under reduced pressure to give compound **6** without further purification (65 mg, 51% yield, two steps). MS (ESI) m/z: $[M + H]^+$, 434.1

N-(3-(4-amino-5-(1-(2-(3-(trifluoromethoxy)phenyl)acetyl)indolin-5-yl)-7H-pyrrolo[2,3-d]pyrimidin-7-yl)propyl)-3-(2-(2-((2-(2,6-dioxopiperidin-3-yl)-1,3-dioxoisoindolin-4-yl)amino)ethoxy)ethoxy)propanamide (7, T2-CRBN): To a solution of 6 (65 mg, 0.15 mmol), 3 (76 mg, 0.15 mmol) in DMF (10 mL) was added HATU (85 mg, 0.22 mmol) and DIPEA (65 μL, 0.45 mmol). The mixture was stirred at RT overnight. The reaction was concentrated and then was purified by reverse phase preparative HPLC to provide compound **7** (**T2-CRBN**) (27 mg, 20% yield). 1H NMR (400 MHz, DMSO-d_6) δ 11.09 (s, 1H), 8.09 (d, J = 12.6 Hz, 2H), 7.89 (s, 1H), 7.53 (t, J = 7.8 Hz, 1H), 7.46 (t, J = 7.7 Hz, 1H), 7.31 (d, J = 14.3 Hz, 4H), 7.23 (dd, J = 17.2, 7.8 Hz, 2H), 7.06 (d, J = 8.6 Hz, 1H), 7.00 (d, J = 7.0 Hz, 1H), 6.55 (s, 1H), 6.04 (s, 1H), 5.03 (dd, J = 12.5, 4.9 Hz, 1H), 4.22 (t, J = 8.1 Hz, 2H), 4.13 (s, 2H), 3.95 (s, 2H), 3.61 – 3.52 (m, 8H), 3.39 (s, 6H), 3.22 (d, J = 8.1 Hz, 2H), 3.02 (d, J = 5.6 Hz, 2H), 2.84 (d, J = 13.3 Hz, 1H), 2.56 (d, J = 16.9 Hz, 1H), 2.29 (t, J = 5.9 Hz, 2H), 2.00 (s, 1H), 1.88 (d, J = 6.5 Hz, 2H). MS (ESI) m/z: $[M + H]^+$, 926.3

(3'R,4'S,5'R)-N-(9-((3-(4-amino-5-(1-(2-(3-(trifluoromethoxy)phenyl)acetyl)indolin-5-yl)-7H-pyrrolo[2,3-d]pyrimidin-7-yl)propyl)amino)-9-oxononyl)-6''-chloro-4'-(3-chloro-2-fluorophenyl)-2''-oxodispiro[cyclohexane-1,2'-pyrrolidine-3',3''-indoline]-5'-carboxamide (10, T2-MDM2): To a solution of **3** (50 mg, 0.1 mmol), 9-((tert-butoxycarbonyl)amino)nonanoic acid (33 mg, 0.12 mmol) in DMF (5 mL) was added HATU (57 mg, 0.15 mmol) and DIPEA (43 µL, 0.3 mmol). The mixture was stirred at RT overnight and was concentrated and then was purified by silica gel flash column chromatography to give the title compound. The compound was dissolved in DCM (2 mL) and TFA (0.5 mL). The mixture was stirred at RT for 1 hour before it was concentrated under reduced pressure to give compound **8** without further purification (30 mg, 46% yield, two steps). ^{1}H NMR (400 MHz, DMSO-d_6) δ 8.54 – 8.46 (m, 1H), 7.92 (s, 5H), 7.70 (d, *J* = 10.6 Hz, 1H), 7.50 – 7.44 (m, 1H), 7.31 (d, *J* = 12.4 Hz, 2H), 7.26 (s, 2H), 4.24 (s, 2H), 3.98 (s, 1H), 3.09 (d, *J* = 4.4 Hz, 2H), 3.03 (d, *J* = 5.5 Hz, 2H), 2.72 (s, 2H), 2.03 (t, *J* = 6.7 Hz, 2H), 1.97 – 1.85 (m, 2H), 1.50 (d, *J* = 6.9 Hz, 2H), 1.45 (s, 2H), 1.28 – 1.24 (m, 8H), 1.23 (s, 4H). MS (ESI) m/z: [M + H]$^+$, 666.3

Compound **9** was prepared according to reported procedures in the literature.[1] Compound **9** (20 mg, 0.043 mmol) and HATU (25 mg, 0.068 mmol) in DMF (10 mL) was added compound **8** (33 mg, 0.05 mmol) and DIPEA (20 µL, 0.135 mmol). The mixture was stirred at RT overnight. The reaction was concentrated and then was purified by reverse phase preparative HPLC to provide compound **10** (**T2-MDM2**) (17 mg, 35% yield) MS (ESI) m/z: [M + H]$^+$, 1110.4

2-(adamantan-1-yl)-N-(3-(4-amino-5-(1-(2-(3-(trifluoromethoxy)phenyl)acetyl)indolin-5-yl)-7H-pyrrolo[2,3-d]pyrimidin-7-yl)propyl)acetamide (11, T2-Ada): To a solution of **3** (20 mg, 0.039 mmol), 2-(adamantan-1-yl)acetic acid (11 mg, 0.058 mmol) in DMF (2 mL) was added HATU (22 mg, 0.058 mmol) and DIPEA (20 µL, 0.135 mmol). The mixture was stirred at RT overnight. The reaction was concentrated and then was purified by reverse phase preparative HPLC to provide compound **11** (**T2-Ada**) (5.6 mg, 21% yield). ^{1}H NMR (400 MHz, DMSO-d_6) δ 8.22 (s, 1H), 7.97 (s, 1H), 7.68 (s, 2H), 7.44 (s, 2H), 7.27 (s, 6H), 4.16 (s, 2H), 3.91 (s, 2H), 1.87 (s, 4H), 1.78 (s, 2H), 1.60 (s, 5H), 1.52 (s, 12H), 1.22 (s, 2H). MS (ESI) m/z: [M + H]$^+$, 687.3

10-oxo-10-(4-(5-phenyl-4,5-dihydro-1H-pyrazole-1-carbonyl)piperidin-1-yl)decanoic acid (13): Compound **12** was prepared according to reported procedures in the literature.[2] To a solution of 10-(tert-butoxy)-10-oxodecanoic acid (10 mg, 0.042 mmol), **12** (9 mg, 0.035 mmol) in 2-methyltetrahydrofuran (3 mL) was added HATU (17 mg, 0.045 mmol) and DIPEA (15 µL, 0.1 mmol). The mixture was stirred at room temperature overnight. The reaction was concentrated and then was purified by silica gel flash column chromatography to give the intermediate.

The above intermediate was dissolved in DCM (2 mL) and TFA (0.5 mL) and the solution was stirred at RT for 5 hours before it was concentrated under reduced pressure to give title compound without further purification (10 mg, 61% yield, two steps). MS (ESI) m/z: [M + H]$^+$, 442.2

(2S,4R)-1-((2S)-3,3-dimethyl-2-(10-oxo-10-(4-(5-phenyl-4,5-dihydro-1H-pyrazole-1-carbonyl)piperidin-1-yl)decanamido)butanoyl)-4-hydroxy-N-((S)-1-(4-(4-methylthiazol-5-yl)phenyl)ethyl)pyrrolidine-2-carboxamide (14, T3-VHL): Compound **14** was prepared using the procedure described for the synthesis of compound **5** by using compound **13** instead of compound **4**. Yield 23%. ^{1}H NMR (400 MHz, DMSO-d_6) δ 9.39 (s, 1H), 9.00 (s, 1H), 8.61 (s, 1H), 8.39 (s, 1H), 8.18 (d, *J* = 15.1 Hz, 2H), 7.79 (s, 1H), 7.47 – 7.32 (m, 5H), 7.24 (s, 1H), 7.11 (s, 1H), 5.30 (s, 1H), 5.11 (s, 1H), 4.93 (s, 2H), 4.52 (s, 1H), 4.29 (s, 1H), 3.87 (s, 2H), 3.59 (s, 1H), 2.82 (s, 2H), 2.67 (d, *J* = 20.1 Hz, 2H), 2.47 (s, 3H), 2.28 (s, 3H), 2.12 (s, 1H), 2.02 (s, 1H), 1.81 (s, 2H), 1.74 (s, 6H), 1.47 (s, 5H), 1.38 (s, 3H), 1.25 (s, 8H), 0.94 (s, 9H). MS (ESI) m/z: [M + H]$^+$, 868.5

2-(2,6-dioxopiperidin-3-yl)-4-((2-(2-(3-oxo-3-(4-(5-phenyl-4,5-dihydro-1H-pyrazole-1-carbonyl)piperidin-1-yl)propoxy)ethoxy)ethyl)amino)isoindoline-1,3-dione (15, T3-CRBN): To a solution of **6** (18 mg, 0.042 mmol), **12** (9 mg, 0.035 mmol) in 2-methyltetrahydrofuran (3 mL) was added HATU (17 mg, 0.045 mmol) and DIPEA (15 µL, 0.1 mmol). The mixture was stirred at room temperature overnight. The reaction was concentrated and then was purified by reverse phase preparative HPLC to give the title compound (9.8 mg, 42% yield). ^{1}H NMR (400 MHz, DMSO-d_6) δ 11.10 (d, *J* = 10.0 Hz, 1H), 7.88 (d, *J* = 53.4 Hz, 1H), 7.57 (s, 3H), 7.37 (s, 2H), 7.14 (d, *J* = 7.6 Hz, 1H), 7.05 – 6.93 (m, 2H), 6.60 (s, 1H), 5.04 (d, *J* = 9.3 Hz, 1H), 4.36 (s, 1H), 3.87 (s, 1H), 3.61 (s, 4H), 3.57 – 3.43 (m, 8H), 3.04 (s, 2H), 2.87 (s, 2H), 2.57 (d, *J* = 15.8 Hz, 2H), 2.03 (s, 1H), 1.72 (s, 2H), 1.58 (s, 2H), 1.37 (s, 2H). MS (ESI) m/z: [M + H]$^+$, 673.3

(3'R,4'S,5'R)-6''-chloro-4'-(3-chloro-2-fluorophenyl)-2''-oxo-N-(2-(2-(3-oxo-3-(4-(5-phenyl-4,5-dihydro-1H-pyrazole-1-carbonyl)piperidin-1-yl)propoxy)ethoxy)ethyl)dispiro[cyclohexane-1,2'-pyrrolidine-3',3''-indoline]-5'-carboxamide (17, T3-MDM2): Compound **17** was prepared using the procedure described for the synthesis of **14** by using 2,2-dimethyl-4-oxo-3,8,11-trioxa-5-azatetradecan-14-oic acid instead of 10-(tert-butoxy)-10-oxodecanoic acid. Yield 23%. ^{1}H NMR (400 MHz, DMSO-d_6) δ 10.52 (s, 1H), 8.17 (s, 1H), 8.04 (s, 1H), 7.57 (s, 1H), 7.37 (s, 1H), 7.30 (s, 1H), 7.25 – 7.11 (m, 2H), 7.08 (d, J = 7.7 Hz, 1H), 7.04 – 6.94 (m, 1H), 6.86 (s, 1H), 6.65 (s, 1H), 4.52 (d, J = 8.8 Hz, 1H), 4.39 (d, J = 9.0 Hz, 1H), 3.90 (s, 2H), 3.63 (s, 2H), 3.50 (s, 4H), 2.70 (s, 2H), 2.01 (s, 2H), 1.89 – 1.67 (m, 4H), 1.56 (s, 4H), 1.53 (s, 2H), 1.22 (s, 2H), 0.86 (t, J = 6.4 Hz, 10H). MS (ESI) m/z: [M + H]$^+$, 861.3

(1-(2-aminoethyl)piperidin-4-yl)(5-phenyl-4,5-dihydro-1H-pyrazol-1-yl)methanone (18): Compound **12** (50 mg, 0.19 mmol) and tert-butyl (2-bromoethyl)carbamate (65 mg, 0.29 mmol) were dissolved in DMF (5 mL). The solution was added K_2CO_3 (55 mg, 0.4 mmol) and the mixture was stirred at RT overnight before it was quenched with water. The resulting mixture was extracted with DCM (3×100 mL), the combined organic phases were dried over anhydrous Na_2SO_4 and concentrated under reduced pressure. The residue was purified by silica gel flash column chromatography to give the product. The product was dissolved in DCM (3 mL) and the solution was slowly added 3 N HCl in methanol (1 mL) in an ice bath. Then the solution was stirred at RT for 2 hours. The mixture was filtered and the solid was dried under vacuum to give the title compound (45 mg, 77% yield, two steps). MS (ESI) m/z: [M + H]$^+$, 301.2

2-(adamantan-1-yl)-N-(2-(4-(5-phenyl-4,5-dihydro-1H-pyrazole-1-carbonyl)piperidin-1-yl)ethyl)acetamide (19, T3-Ada): Compound **19** was prepared using the procedure described for the synthesis of **11** by using compound **18** instead of compound **3**. Yield: 23%. ^{1}H NMR (400 MHz, DMSO-d_6) δ 8.13 (s, 1H), 7.55 (s, 1H), 7.29 (d, J = 7.2 Hz, 1H), 7.22 (d, J = 7.5 Hz, 1H), 7.18 (s, 1H), 7.07 (d, J = 7.2 Hz, 2H), 5.27 (d, J = 8.3 Hz, 1H), 3.12 (d, J = 5.4 Hz, 2H), 2.91 (d, J = 9.2 Hz, 2H), 2.36 (s, 2H), 2.03 (s, 2H), 1.87 (s, 3H), 1.77 (d, J = 14.3 Hz, 3H), 1.61 (d, J = 9.6 Hz, 4H), 1.53 (s, 11H). MS (ESI) m/z: [M + H]$^+$, 477.3

N-(3-(4-amino-5-(1-(2-(3-(trifluoromethoxy)phenyl)acetyl)indolin-5-yl)-7H-pyrrolo[2,3-d]pyrimidin-7-yl)propyl)-3-(5,5-difluoro-7,9-dimethyl-5H-5l4,6l4-dipyrrolo[1,2-c:2',1'-f][1,3,2]diazaborinin-3-yl)propenamide (T2-488): To a solution of **3** (8 mg, 0.015 mmol) in DMF (2 mL) was added BDP FL NHS ester (5 mg, 0.013 mmol) and DIPEA (8 µL, 0.05 mmol). The mixture was stirred at room temperature for 5 hours. The reaction was concentrated and then was purified by reverse phase preparative HPLC to give the title compound (8.8 mg, 86% yield). Yield: 86%. ^{1}H NMR (400 MHz, DMSO-d_6) δ 8.20 (s, 1H), 8.15 – 8.04 (m, 2H), 8.00 (s, 1H), 7.64 (s, 1H), 7.47 – 7.41 (m, 1H), 7.29 (t, J = 8.3 Hz, 3H), 7.20 (d, J = 8.7 Hz, 1H), 7.04 (s, 1H), 6.32 (s, 1H), 6.25 (s, 1H), 4.19 (d, J = 8.0 Hz, 2H), 4.12 (s, 2H), 3.93 (s, 2H), 3.20 (s, 2H), 3.05 (s, 4H), 2.46 (s, 6H), 2.42 (s, 2H), 2.21 (s, 2H), 1.88 (s, 2H). ^{13}C NMR (100 MHz, DMSO-d_6) δ 171.28, 168.91, 159.58, 158.22, 157.66, 151.95, 150.49, 148.68, 144.52, 142.20, 138.46, 134.89, 133.42, 133.28, 130.41, 130.32, 129.46, 129.36, 127.58, 125.37, 124.73 (q, J = 208 Hz), 122.69, 120.71, 119.44, 116.99, 116.62, 115.46, 100.41, 48.30, 42.07, 41.68, 36.52, 34.72, 34.23, 30.26, 27.93, 24.45, 14.94, 11.43. MS (ESI) m/z: [M + H]$^+$, 785.3

N-(3-(4-amino-5-(1-(2-(3-(trifluoromethoxy)phenyl)acetyl)indolin-5-yl)-7H-pyrrolo[2,3-d]pyrimidin-7-yl)propyl)-3-(5,5-difluoro-9-(1H-pyrrol-2-yl)-5H-4l4,5l4-dipyrrolo[1,2-c:2',1'-f][1,3,2]diazaborinin-3-yl)propenamide (T2-590): Compound **T2-590** was prepared using the procedure described for the synthesis of **T2-488** by using BDP 576/589 NHS ester instead of BDP FL NHS ester. Yield: 71%. ^{1}H NMR (400 MHz, $CDCl_3$) δ 10.42 (s, 1H), 8.30 (d, J = 7.9 Hz, 1H), 8.26 (s, 1H), 7.39 (t, J = 7.8 Hz, 1H), 7.29 (s, 1H), 7.22 – 7.13 (m, 2H), 7.10 (s, 1H), 7.03 (d, J = 10.8 Hz, 2H), 6.96 (d, J = 7.6 Hz, 2H), 6.90 (s, 1H), 6.85 (s, 1H), 6.82 (s, 1H), 6.33 (d, J = 9.8 Hz, 2H), 5.42 (s, 2H), 5.29 (s, 1H), 4.17 – 4.11 (m, 2H), 3.85 (s, 2H), 3.38 (t, J = 7.2 Hz, 2H), 3.24 (t, J = 7.9 Hz, 2H), 3.14 (d, J = 4.0 Hz, 2H), 2.72 (t, J = 7.0 Hz, 2H), 1.96 (s, 2H), 1.25 (s, 1H). ^{13}C NMR (100 MHz, $CDCl_3$) δ 171.85, 168.25, 156.86, 155.18, 151.43, 150.50, 149.45, 142.25, 137.33, 136.24, 133.44, 132.17, 131.57, 130.02, 127.61, 127.15 (q, J = 206 Hz), 125.81, 125.01, 123.25, 122.66, 121.86, 120.36, 119.52, 117.82, 117.50, 116.65, 116.58, 111.45, 100.78, 48.38, 42.84, 41.43, 36.06, 35.63, 30.17, 28.06, 24.80. MS (ESI) m/z: [M + H]$^+$, 822.3
