# Supplementary material for: Development of a RIPK1 degrader to enhance antitumor immunity
Source: Nat Commun. 2024 Dec 16;15:10683. doi: 10.1038/s41467-024-55006-2 (PMC11649918; doi:10.1038/s41467-024-55006-2)
Supplement: Supplementary file 2 — Reporting Summary [file 41467_2024_55006_MOESM2_ESM.pdf]

nature portfolio

Corresponding author(s): Jin Wang
Last updated by author(s): Oct 26, 2024

Reporting Summary

Nature Portfolio wishes to improve the reproducibility of the work that we publish. This form provides structure for consistency and transparency in reporting. For further information on Nature Portfolio policies, see our Editorial Policies and the Editorial Policy Checklist.

Statistics

For all statistical analyses, confirm that the following items are present in the figure legend, table legend, main text, or Methods section.

n/a	Confirmed	
☐	☒	The exact sample size (n) for each experimental group/condition, given as a discrete number and unit of measurement
☒	☐	A statement on whether measurements were taken from distinct samples or whether the same sample was measured repeatedly
☐	☒	The statistical test(s) used AND whether they are one- or two-sided<br>*Only common tests should be described solely by name; describe more complex techniques in the Methods section.*
☒	☐	A description of all covariates tested
☒	☐	A description of any assumptions or corrections, such as tests of normality and adjustment for multiple comparisons
☐	☒	A full description of the statistical parameters including central tendency (e.g. means) or other basic estimates (e.g. regression coefficient) AND variation (e.g. standard deviation) or associated estimates of uncertainty (e.g. confidence intervals)
☐	☒	For null hypothesis testing, the test statistic (e.g. F, t, r) with confidence intervals, effect sizes, degrees of freedom and P value noted<br>*Give P values as exact values whenever suitable.*
☒	☐	For Bayesian analysis, information on the choice of priors and Markov chain Monte Carlo settings
☒	☐	For hierarchical and complex designs, identification of the appropriate level for tests and full reporting of outcomes
☒	☐	Estimates of effect sizes (e.g. Cohen's d, Pearson's r), indicating how they were calculated

Our web collection on statistics for biologists contains articles on many of the points above.

Software and code

Policy information about availability of computer code

Data collection	Nuclear magnetic resonance (NMR) data was collected using VnmrJ Software on a Varian Palo Alto 400MHz NMR spectrometer. Mass spectra were acquired using OpenLab Chemstation on an Agilent 1260 Infinity LC/MS System. Molecular docking was performed at Schrödinger 2022-1 software. Proteomics data was collected using Xcalibur software on a Thermofisher Orbitrap Fusion™ Lumos™ Tribrid™ Mass Spectrometer.<br>Flow cytometry data were collected using BD FACSDiva software on a BD LSR-II. Western blot imaging was performed with the KwikQuant Digital Western Blot Detection System (D1001, Kindle Biosciences). Immunofluorescence (IF) and immunohistochemistry (IHC) tissue slides were scanned using the Zeiss LSM780 confocal microscope.For the TR-FRET assay, fluorescence emission ratios (I520 nm/I490 nm) were measured using a BioTek Synergy H1 microplate reader, with a 340-nm excitation filter, a 100-µs delay, and a 200-µs integration time. For the Nano-BRET assay, donor emission at 450 nm and acceptor emission at 610 nm were measured on a BioTek Synergy H1 plate reader equipped with a filter cube set at 450/80 and 610 LP. Extracellular ATP assay luminescence signals were recorded using the BioTek Synergy H1 microplate reader.
Data analysis	NMR data was analyzed with MestReNova. Proteomics data was analyzed with Proteome Discoverer 2.4. Flow cytometry data were analyzed with FlowJo (version 10.9.0). Bar graphs and statistical tests were generated using GraphPad Prism (version 9.3.1). ImageJ (version 2.1.0/1.53c) was employed for quantifying Western blot band intensity.

For manuscripts utilizing custom algorithms or software that are central to the research but not yet described in published literature, software must be made available to editors and reviewers. We strongly encourage code deposition in a community repository (e.g. GitHub). See the Nature Portfolio guidelines for submitting code & software for further information.

Data

Policy information about availability of data

All manuscripts must include a data availability statement. This statement should provide the following information, where applicable:

- Accession codes, unique identifiers, or web links for publicly available datasets
- A description of any restrictions on data availability
- For clinical datasets or third party data, please ensure that the statement adheres to our policy

Accession codes, unique identifiers, or web links for publicly available datasets

Research involving human participants, their data, or biological material

Policy information about studies with human participants or human data. See also policy information about sex, gender (identity/presentation), and sexual orientation and race, ethnicity and racism.

Reporting on sex and gender	N/A
Reporting on race, ethnicity, or other socially relevant groupings	N/A
Population characteristics	N/A
Recruitment	N/A
Ethics oversight	N/A

Note that full information on the approval of the study protocol must also be provided in the manuscript.

Field-specific reporting

Please select the one below that is the best fit for your research. If you are not sure, read the appropriate sections before making your selection.

[x] Life sciences [ ] Behavioural & social sciences [ ] Ecological, evolutionary & environmental sciences

For a reference copy of the document with all sections, see nature.com/documents/nr-reporting-summary-flat.pdf

Life sciences study design

All studies must disclose on these points even when the disclosure is negative.

Sample size	Western blots are usually ran with duplicates. TR-FRET and NanoBRET target engagement assays are performed with triplicates. Cell death assays were performed with at least triplicates. Based on literature and previous studies, these sample sizes are sufficient.
Data exclusions	No data exclusion
Replication	All the experimental findings have been reproduced.
Randomization	All mice were allocated into groups randomly before starting drug treatment.
Blinding	No bliding

Reporting for specific materials, systems and methods

We require information from authors about some types of materials, experimental systems and methods used in many studies. Here, indicate whether each material, system or method listed is relevant to your study. If you are not sure if a list item applies to your research, read the appropriate section before selecting a response.

Materials & experimental systems

n/a	Involved in the study	
[ ]	[x]	Antibodies
[ ]	[x]	Eukaryotic cell lines
[x]	[ ]	Palaeontology and archaeology
[ ]	[x]	Animals and other organisms
[x]	[ ]	Clinical data
[x]	[ ]	Dual use research of concern
[x]	[ ]	Plants

Methods

n/a	Involved in the study	
[x]	[ ]	ChIP-seq
[ ]	[x]	Flow cytometry
[x]	[ ]	MRI-based neuroimaging

Antibodies

Antibodies used	For flow cytometry: The anti-mouse antibodies were diluted as follows: CD45-APC750 (103153, Biolegend, clone 30-F11, 1:200), CD3e-APC (100311,Biolegend, clone 145-2C11,1:200), CD4-BV650 (100469,Biolegend, clone GK1.5,1:200), CD8-PercpCy5.5 (155013,Biolegend, clone 53-6.7,1:200), PD1-PE (114117, Biolegend, clone RMP1-14,1:200), IA/I-E-APC (107613, Biolegend, clone M5/114.15.2,1:200), CD11c-PE (117307,Biolegend, clone N418,1:200), CD11b-PercpCy5.5 (101227,Biolegend, clone M1/70,1:200), Ly6C-AF700 (128023,Biolegend, clone HK1.4,1:200), F4/80-FITC (123107,Biolegend, clone BM8,1:200), XCR1-BV650 (148220,Biolegend, clone ZET,1:200). For Western blot: anti-RIPK1 (3493, Cell Signaling Technology (CST),1:1000), anti-cleaved caspase 3 (9661, CST,1:1000), anti-cleaved caspase7 (8438, CST.1:1000), anti-cleaved PARP (5625,CST,1:1000), anti-HMGB1 (3935,CST, 1:1000), anti-calreticulin (12238,CST,1:1000), anti-β-actin (4970, CST,1:1000). For IF and IHC: cleaved caspase 3 (9661, CST,1:100), cleaved caspase 7 (8438, CST,1:100), CD8 (14-0808-82, eBioscience,1:100), CD4 (NBP1-19371, Novus Biologicals,1:100), FOXP3 (NB100-39002, Novus Biologicals,1:100), F4/80 (NB600-404, Novus Biologicals,1:100). For in vivo antibody administration: anti-PD1 (BE0146, BioXcell) and anti-CD8 (BE0004, BioXcell).
Validation	All the antibodies used in this study are commercially and have been validated by their manufacturers. This infromation is available on their website.

Eukaryotic cell lines

Policy information about cell lines and Sex and Gender in Research

Cell line source(s)	Human and mouse hematopoietic cell lines, namely Jurkat, Ramos, THP1, U937, TK1, and A20, and mouse melanoma B16F10 cell lines, were procured from ATCC. MC38 colon carcinoma and H2023 lung carcinoma cells were provided by Dr. Weiyi Peng. A375 melanoma cells were acquired from the Cell Core at the MD Anderson Cancer Center. Human breast cancer cells MDA-MB-231 and BT474 were obtained from Baylor College of Medicine Cell Core, whereas mouse breast carcinoma 4T1 cells were a gift from Dr. Xiang Zhang.
Authentication	None of the cell lines used were authenticated.
Mycoplasma contamination	B16F10 cell line was tested negative for mycoplasma contamination.
Commonly misidentified lines (See ICLAC register)	N/A

Animals and other research organisms

Policy information about studies involving animals; ARRIVE guidelines recommended for reporting animal research, and Sex and Gender in Research

Laboratory animals	Female C57BL/6J mice (6-weeks-old) were were ordered from Jackson Labs. Mice were housed 2-4 per cage in an American Animal Association Laboratory Animal Care accredited facility and maintained under standard conditions of temperature (22 ± 2°C), relative humidity (50%) and light and dark cycle (12/12 hours), and had access to food and water ad libitum. Mice were allowed to acclimate to their environment for one week before experiment.
Wild animals	No wild animals were used in the study.
Reporting on sex	We didn't consider mice gender in the study.
Field-collected samples	This study did not involve field-collected samples.
Ethics oversight	We have complied with all relevant ethical regulations for animal testing and research. All the animal experiments were approved by the Institutional Animal Care and Use Committee (IACUC) at Baylor College of Medicine.

Note that full information on the approval of the study protocol must also be provided in the manuscript.

Plants

Seed stocks	N/A
Novel plant genotypes	N/A
Authentication	N/A

Flow Cytometry

Plots

Confirm that:

- [x] The axis labels state the marker and fluorochrome used (e.g. CD4-FITC).
- [x] The axis scales are clearly visible. Include numbers along axes only for bottom left plot of group (a 'group' is an analysis of identical markers).
- [x] All plots are contour plots with outliers or pseudocolor plots.
- [x] A numerical value for number of cells or percentage (with statistics) is provided.

Methodology

Sample preparation	B16F10 cells (2×10^5) were seeded onto six-well plates and treated as specified for 72 hours at 37°C. Treated and untreated cells were harvested, washed with PBS, and resuspended in 100 µl of binding buffer (556547, BD Biosciences). Subsequently, cells were stained with PI (50 µg/ml) and FITC-conjugated Annexin V (10 mg/ml) for 15 minutes at room temperature in the dark. After adding another 400 µl of binding buffer, the cells were ready for analysis.<br>B16F10 tumors were weighed, mechanically diced, and digested with liberase (2 mg/mL, 05401020001, Roche) and DNase I (50 µg/mL, 11284932001, Sigma-Aldrich) at 37°C for 30 minutes with rotation. Single-cell suspensions were obtained by filtering the digested tissues through a 45 µm strainer, after which erythrocytes were removed using 1x RBC lysis buffer (420301, Biolegend). To stain the cell surface markers of tumor-infiltrating lymphocytes (TILs), single-cell suspensions were blocked with anti-mouse CD16/32 (156603, BioLegend) for 10 minutes on ice, and then incubated with fluorochrome-labeled antibodies diluted with staining buffer (1:50, PBS, 2% FBS, 0.1% EDTA) for 30 minutes on ice in the dark. Dead cells were excluded using DAPI (1:1,000, BioLegend). After washing, cells were resuspended in 300-500 µL staining buffer for flow cytometry analysis.
Instrument	Flow cytometry data was collected using BD FACSDiva™ software on BD-LSR II Flow cytometer.
Software	BD FACSDiva™ software was used for data collection. Flow Jo v10.0 was used for data analysis.
Cell population abundance	N/A
Gating strategy	Cells were sequentially gated as lymphocytes (based on FSC-A, SSC-A), single cells (based on FSC-H, FSC-A and SSC-A, SSC-W), live cells (gated as DAPI negative), and interested cell types (e.g., CD3+ total T cells). Positive gates were set based on unstained controls.

- [x] Tick this box to confirm that a figure exemplifying the gating strategy is provided in the Supplementary Information.
